# Thermal Pain Thresholds Are Significantly Associated with Plasma Proteins of the Immune System in Chronic Widespread Pain—An Exploratory Pilot Study Using Multivariate and Network Analyses

**DOI:** 10.3390/jcm10163652

**Published:** 2021-08-18

**Authors:** Björn Gerdle, Karin Wåhlén, Torsten Gordh, Bijar Ghafouri

**Affiliations:** 1 Pain and Rehabilitation Centre, Department of Health, Medicine and Caring Sciences, Linköping University, SE-581 85 Linköping, Sweden; Karin.wahlen@liu.se (K.W.); bijar.ghafouri@liu.se (B.G.); 2 Department of Surgical Sciences, Uppsala University, SE-751 85 Uppsala, Sweden; Torsten.Gordh@surgsci.uu.se or

**Keywords:** biomarker, cold pain threshold, CPT, fibromyalgia, heat pain threshold, HPT, hyperalgesia, pain, sensitivity, widespread pain, proteomics

## Abstract

Chronic widespread pain (CWP), including fibromyalgia (FM), is characterized by generalized musculoskeletal pain. An important clinical feature is widespread increased pain sensitivity such as lowered pain thresholds for different stimuli such as heat (HPT) and cold (CPT). There is a growing interest in investigating the activated neurobiological mechanisms in CWP. This explorative proteomic study investigates the multivariate correlation pattern between plasma and muscle proteins and thermal pain thresholds in CWP and in healthy controls (CON). In addition, we analysed whether the important proteins and their networks for CPT and HPT differed between CWP and CON. We used a proteomic approach and analysed plasma and muscle proteins from women with CWP (*n* = 15) and CON (*n* = 23). The associations between the proteins and CPT/HPT were analysed using orthogonal partial least square (OPLS). The protein–protein association networks for the important proteins for the two thermal pain thresholds were analysed using STRING database. CWP had lowered pain thresholds for thermal stimulus. These levels were generally not related to the included clinical variables except in CWP for HPT. Highly interacting proteins mainly from plasma showed strong significant associations with CPT and HPT both in CWP and in CON. Marked differences in the important proteins for the two thermal pain thresholds were noted between CWP and CON; more complex patterns emerged in CWP. The important proteins were part of the immune system (acute phase proteins, complement factors, and immunoglobulin factors) or known to interact with the immune system. As expected, CWP had lowered pain thresholds for thermal stimulus. Although different proteins were important in the two groups, there were similarities. For example, proteins related to the host defence/immunity such as acute phase proteins, complement factors, immunoglobulin factors, and cytokines/chemokines (although not in CON for CPT) were important habitual/tonic factors for thermal pain thresholds. The fact that peripheral proteins contribute to thermal pain thresholds does not exclude that central factors also contribute and that complex interactions between peripheral and central factors determine the registered pain thresholds in CWP.

## 1. Introduction

Generalized musculoskeletal pain (i.e., chronic widespread pain; CWP) has a high population prevalence (5–10%) with a female predominance [1,2,3]. CWP is associated with increased prevalence of comorbidities such as depressive and anxiety symptoms, sleep problems, and cognitive difficulties [4,5,6]. Thus, CWP as well as other chronic pain conditions have negative consequences that lead to significant suffering for patients and their families and high socioeconomic costs [7].

Assessments, as well as design and choice of treatments, are hampered by lack of valid biomarkers [8,9]. Potential markers of neurobiological mechanisms in CWP including fibromyalgia (FM) such as cytokines/chemokines, lipids, and metabolites in blood, saliva, muscles, and cerebrospinal fluid are increasingly reported [10,11,12,13,14,15,16,17,18]. Omic methods are gaining increasing support as pain seemingly involves a myriad of molecular changes [19]. The proteome of a tissue regulates biological processes and integrates the effects of genes with environmental factors, comorbidities, behaviours, age, and drugs [19,20,21]. Compared to genome and transcriptome studies, investigating the proteome is more complex, including the choice of statistical methods [22]. We and others have reported marked significant differences both in blood and in muscle proteomes between CWP/FM and healthy controls [10,23,24,25,26]. Moreover, both muscle and plasma protein patterns show strong significant correlations with pain intensity [25,27,28].

Recently nociplastic pain was introduced as a pain mechanistic descriptor (IASP definition: ”Pain that arises from altered nociception despite no clear evidence of actual or threatened tissue damage causing the activation of peripheral nociceptors or evidence for disease or lesion of the somatosensory system causing the pain.” Source: https://www.iasp-pain.org/Education/Content.aspx?ItemNumber=1698#Nociplasticpain; access date: 1 June 2021); FM (a subgroup of CWP) is classified as a nociplastic pain condition [29]. Although validated and definite clinical criteria for nociplastic pain are still lacking, it can be reasonably assumed that these criteria would at the minimum include increased spatial distribution of pain and increased pain sensitivity [30,31,32]. CWP including FM is generally associated with increased pain sensitivity (i.e., lowered pain thresholds) [33]. Pain threshold is defined as when an acute stimulus becomes painful; the stimulus both activates peripheral nociceptors and the central nervous system (CNS). Pressure pain threshold (PPT) correlates significantly with certain plasma proteins obtained from proteomic analysis (i.e., proteins typically at nano and micro molar levels) and with proteins from targeted analysis of a panel of 92 inflammation-related substances (cytokines, chemokines, and growth factors—i.e., small molecules typically at picomolar levels) in CWP [17,34]. The strongest regression was obtained using proteomics. Recently, we reported that plasma proteins correlated with PPT in an FM cohort [25].

In addition to PPT, pain thresholds for cold (CPT) and heat (HPT) are used to develop a nuanced picture of the pain sensitivity. As both muscle and plasma proteins influence PPT in CWP, it may be appropriate to examine them together in relation to CPT and HPT. This study was further motivated by the fact that peripheral molecular mechanisms (in plasma and in muscle) underlying heat and cold pain thresholds are mainly lacking [35]. The need to investigate clinical variables such as pain thresholds in relation to peripheral biomarkers has been emphasized [36]. In chronic pain conditions, comorbidities such as psychological distress and obesity are common, and neurobiological alterations associated with these comorbidities may influence pain thresholds. For example, psychological distress and catastrophizing correlate with thermal pain thresholds [37,38].

We formulated three hypotheses: (1) thermal pain thresholds differ between CWP patients and controls; (2) thermal pain thresholds are significantly associated with peripheral protein patterns in blood (plasma) and in muscle; and (3) the important proteins differed between the two groups of subjects. Therefore, this exploratory study investigates the multivariate correlation pattern between thermal pain thresholds (CPT and HPT) and proteins from plasma and muscle in CWP (typically with lowered pain thresholds) and in healthy controls. We analysed whether the important proteins and their networks involved in the two thresholds differed between the two groups and to what extent comorbidities influenced the thresholds.

## 2. Materials and Methods

### 2.1. Subjects

**Figure 1:**
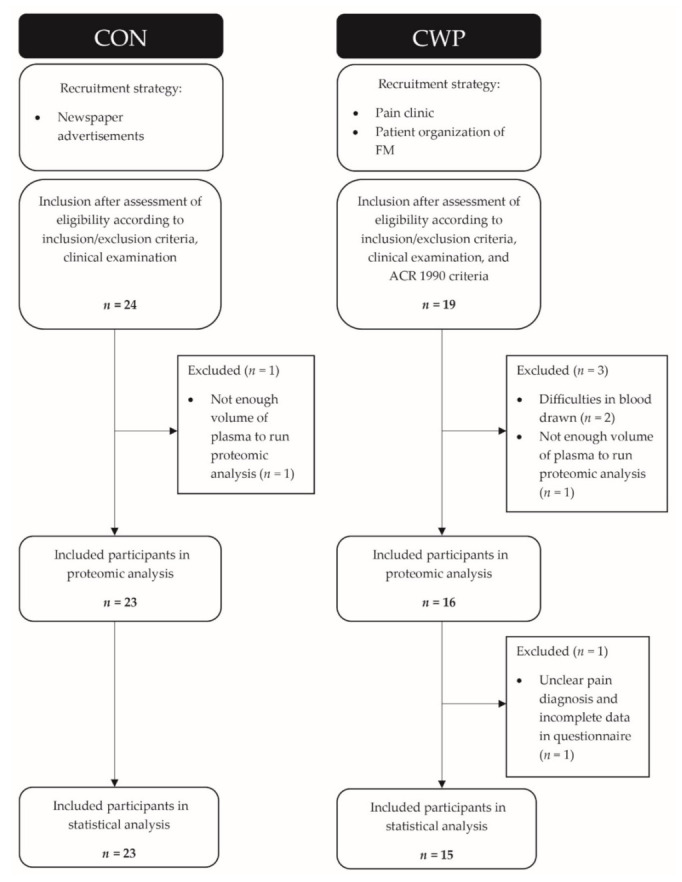
Flow chart of included chronic widespread pain (CWP) patients and healthy controls (CON).

The recruitment of subjects (patients with CWP and healthy controls (CON)) has been described elsewhere [39]. The American College of Rheumatology (ACR) criteria from 1990 was used to classify FM/CWP [6]. The criteria for CWP according to these criteria are chronic pain (>3 months), spinal pain, and pain in at least three out of four body quadrants (or in two contralateral body quadrants). As reported earlier, 13 out of 15 CWP patients also fulfilled the criteria for FM [28]. All subjects were women and none used any anticoagulatory, opioid, or steroidal medication. Exclusion criteria were medical history record of certain musculoskeletal conditions (bursitis, capsulitis, neck trauma, postoperative conditions in neck/shoulder area, spine disorders, tendonitis, rheumatoid arthritis), neurological disease, or systemic or metabolic diseases, malignancy, severe psychiatric illness, pregnancy, and difficulties understanding Swedish. The CON group were recruited through newspaper advertisements. CWP patients were recruited from former female patients at the Pain and Rehabilitation Centre at the University Hospital, Linköping, Sweden or from an organization for FM patients. A total of 19 CWP and 24 CON were initially recruited as reported earlier [10,39,40,41] and this study included 15 CWP patients and 23 CON subjects; see flow chart for details (Figure 1).

The study followed the guidelines in the Declaration of Helsinki and was approved by the Regional Ethical Review Board in Linköping, Sweden (Diarie-nr. M10–08, M233–09, Diarie-nr. 2010/164–32). In agreement with this, all subjects signed a written consent before the start of the study and after receiving verbal and written information about the aims and procedures.

### 2.2. Clinical Variables

All subjects answered a brief health questionnaire. As these data were previously published, we only provide summaries of the patient reported outcome measures (PROM) variables and instruments used.

Age and body mass index: Age (y), weight (kg), and height (m) were registered at the clinical examination. Body mass index (BMI) was calculated as weight/height^2^ (kg/m^2^).

Pain intensity: Each subject rated their pain intensity in the neck, the shoulders, and the whole body using an 11-grade (0–10) numeric rating scale (NRS) (endpoints: zero = no pain at all and 10 = worst possible pain) [42].

Psychological distress: The Hospital Anxiety and Depression Scale (HADS) was used for the self-assessments of anxiety and depression symptoms [43]. In agreement with a large psychometric analysis, a total score of HADS (denoted HADS; possible range 0–42)—including both the anxiety and depression scores—was used as a measure of psychological distress [44].

Catastrophizing: Catastrophizing aspects, i.e., rumination, magnification, and helplessness were measured using the Pain Catastrophizing Scale (PCS) [45]. The total PCS score was used (maximum score: 52).

Quality of life: This was measured using the Quality-of-Life Scale (QOLS) [46]. Sixteen items (each measured on a seven-point satisfaction scale) are added to a total score (possible range: 16–112). A lower score reflects lower satisfaction.

### 2.3. Pain Thresholds for Cold and Heat

Pain thresholds for cold (CPT) and heat (HPT) were determined using a modular sensory analyser (MSA) from Somedic, Hörby, Sweden; see earlier studies for details [47,48]. A skilled research nurse performed all tests. The testing was conducted over the upper part of the trapezius muscle (bilaterally approximately midway on a line between C7 and the acromion). The two main reasons for measuring CPT and HPT over the trapezius muscle were: (1) patients have pain in the neck-shoulder region as part of their CWP and (2) biopsies were taken from the trapezius muscle. A structured protocol according to the Marstock method was applied for all tests [49]. The thermode had a 25 × 50 mm stimulating surface consisting of Peltier elements and a temperature change range of 1 °C/s. The subjects sat comfortably in a quiet room with an ambient temperature (approximately 22 °C). Registrations of CPT and HPT were made a few days before the blood sampling.

### 2.4. Proteins and Other Biochemical Substances

We analysed proteins from plasma and muscle in relation to the thermal pain thresholds in CWP and CON.

#### 2.4.1. Sample Collection

##### Blood Sampling

Venous blood samples were collected using EDTA tubes. A washout period of seven days for nonsteroidal anti-inflammatory drugs and 12 h for paracetamol medication was used. Plasma was extracted and prepared as previously described [10].

##### Muscle Biopsy Sampling

The sampling and preparation of muscle biopsies are fully described in our previous studies [23,27]. Briefly, biopsies were sampled from the upper trapezius muscle at the midpoint between the 7th cervical vertebra and the acromion on the most painful side (generally the dominant side) using Monopty BARD microbiopsy instrument (BARD Norden, Helsingborg, Sweden). Immediately after sampling, the muscle tissues were quickly frozen by immersing them in isopentane precooled with dry ice and then stored at −86 °C until analysis. Before analysis, the muscle tissues were heat stabilized using Denator Stabilizer T1 (Denator, Gothenburg, Sweden), placed in a tube containing urea sample buffer solution, homogenized, and prepared as previously described [23,27].

#### 2.4.2. Biochemical Analyses

Both plasma and muscle biopsy samples were used for proteomic analyses. In addition, certain cytokines, chemokines, and growth factors in the plasma samples were identified using a proximal extension assay.

##### Proteomics of Muscle Biopsy and Plasma—Two-Dimensional Gel Electrophoresis (2-DE)

Here, we briefly summarise the 2-DE procedure as the procedure was described in detail earlier [10,23,50]. Proteomic analysis of depleted plasma samples and prepared muscle biopsies was carried out using Ettan IPGphor 3 IEF System (GE Healthcare, Buckinghamshire, UK) (first dimension) and Ettan DALTsix Electrophoresis Unit (Amersham, Pharmacia, Uppsala, Sweden) (second dimension). The fluorescent stain SYPRO Ruby (Bio-Rad Laboratories, Hercules, CA, USA) was applied to plasma protein gels, and silver stain was applied to muscle biopsy gels. The stained protein pattern was visualized using a charge coupled device camera (VersaDoc Imaging system 4000 MP, Bio-Rad). The PDQuest Advanced (v. 8.0.1, Bio-Rad) software was used to analyse and quantify the protein pattern. The amount of protein in a certain spot was assessed as background corrected optical density integrated over all pixels in the spot and expressed as integrated optical density (IOD). The parts per million (ppm) values for all proteins were generated and used for further statistical analysis.

##### Protein Identification

To identify proteins, spots of interest were excised from preparative fluorescently stained plasma and biopsy gels (400 µg of total protein), destained, tryptically digested, and prepared for mass spectrometry (MS) analysis [23]. Protein identification was carried out using two MS instruments: ultrafleXtreme matrix-assisted laser desorption/ionization time-of-flight (MALDI-TOF, Bruker Daltonik GmbH, Bremen, Germany) and nano liquid chromatography system (EASY-nLC, Thermo Scientific, Waltham, MA, USA) with a C18 column (100 mm × 0.75 µm, Agilent Technologies, Santa Clara, CA, USA) coupled to a LTQ Orbitrap Velos Pro mass spectrometer (Thermo Scientific).

##### Database Search

The search strategies for protein identification were described in detail earlier [10,14]. In brief, the acquired MS data from MALDI-TOF analysis were pre-processed using flexAnalysis v. 3.4 (Bruker Daltonik). The major peak list from each processed spectrum was imported into ProteinProspector MS-Fit search engineer (v. 5.14.4), including the Swiss-Prot database v. 2015.3.5 [10,14]. The acquired MS data from the Orbitrap was analysed with MaxQuant v. 1.5.8.3 (Max Planck Institute of Biochemistry, Martinsried, Germany) using the human UniProt/Swiss-Prot database (downloaded 4 April 2017) as reported previously [14].

##### Proximal Extension Assay for Identifying Cytokines, Chemokines, and Growth Factors

A multiplex proximity extension assay (PEA) was used to analyse 92 cytokines, chemokines, and growth factors simultaneously in the plasma samples as described previously [17]. The multiplex PEA was conducted using Proseek Multiplex Inflammation I (Olink Bioscience, Uppsala, Sweden) per the manufacturer’s instructions. The acquired data from the PEA analysis are expressed as normalized protein expression (NPX). The normalized protein expression (NPX) values were used for further statistical analysis.

### 2.5. Statistics

Student’s *t*-test was applied for comparison of group values of background variables and thermal pain thresholds (CPT and HPT) using IBM SPSS (version 24.0; IBM Corporation, Route 100 Somers, New York, USA); *p* < 0.05 was considered significant.

Multivariate data analysis (MVDA) is necessary when analysing omics data [36,51]. We used SIMCA-P+ (version 15.0; Sartorius Stedim Biotech, Umeå, Sweden) for MVDA and applied the recommendations concerning MVDA of omics data [51]. Orthogonal partial least squares (OPLS) regression analysis was used for the regression analyses of HPT and CPT of the trapezius as Y-variable and the proteins and clinical variables as regressors (X-variables) [52]. For detailed descriptions see previous studies: [10,14,23,34]. All variables were mean centred, scaled to unit variance (UV-scaling), and log-transformed if necessary. No multivariate outliers were identified according to the principal component analysis. Variables with variable influence on projection (predictive) value (VIPpred) >1.0 (combined with jack-knifed 95% confidence intervals in the regression coefficients plot not including zero) and with absolute p(corr) ≥ 0.40 were considered significant; p(corr) is the loading of each variable scaled as a correlation coefficient with a standardised range (−1 to +1) [51]. The OPLS analysis was made in two steps. In the first step, all proteins (several hundred) were included in the analysis. In the second step, the 20 proteins with the VIPpred ≥ 1.0 and p(corr) ≥ 0.40 were used in a new OPLS regression provided that the first analysis resulted in a significant component according to the internal rules used in SIMCA-P+ [52]. This article presents the results from the second step. R^2^ describes the goodness of fit and Q^2^ describes goodness of prediction [52]. Cross validated analysis of variance (CV-ANOVA) with a *p* ≤ 0.05 was used to validate the obtained model.

### 2.6. Network Analysis

The protein–protein association network for the important proteins for the two thermal pain thresholds in CWP and CON were separately analysed using the online database Search Tool for Retrieval of Interacting Genes/Proteins (STRING; version 11) [53]. The search settings for the networks were set to: *Homo sapiens* (species); query proteins only (maximum number of protein interactions); minimum interaction score of medium confidence (0.400); and an FDR ≤ 0.001 for classifying the cellular component (CC), molecular function (MF), and biological processes (BP) according to Gene Ontology (GO; http://geneontology.org/docs/ontology-documentation/ access date: 15 March 2021). For each obtained network, PPI enrichment *p*-value and average local clustering coefficient were reported. In the network figure, each protein is represented by a coloured node, and protein–protein interaction and association are represented by an edge visualized as a line. Thicker lines/edges represent higher combined confidence scores.

## 3. Results

### 3.1. Clinical Variables

These data were presented elsewhere for the two groups and are summarized in Table 1 [28,34]. CWP patients reported higher levels of psychological distress and catastrophizing and lower quality of life. As expected, CWP had considerably higher pain intensities, and CON were pain free. CWP were significantly older.

### 3.2. Thermal Pain Thresholds

Significant group differences were found both for CPT and HPT (Table 1). CWP had significantly increased pain sensitivity according to both cold (reported at warmer temperature) and heat (reported at lower temperature).

### 3.3. Clinical Variables as Regressors of CPT and HPT

Neither CPT and HPT in CON nor CPT in CWP exhibited significant regressions, indicating that the clinical variables, including BMI and age, had little or no influence (for details, see Text S1). In contrast, the regression of HPT was significant (R^2^ = 0.67, Q^2^ = 0.41, CV-ANOVA *p*-value: 0.043; one predictive component) and HADS (VIPpred = 1.78; p(corr) = −0.85) and QOLS (VIPpred = 1.60; p(corr) = 0.77) were significant regressors (VIPpred > 1.0), whereas BMI, NRS-shoulders, NRS-neck, PCS, and age were not important (for details, see Text S1).

### 3.4. Proteins as Regressors of CPT and HPT

The proteins from muscle and plasma were used to regress CPT and HPT in CON and CWP.

#### 3.4.1. CPT

Significant regressions of CPT were obtained both in CON (R^2^ = 0.94, Q^2^ = 0.76, CV-ANOVA *p*-value: 0.005; one predictive and three orthogonal components) and in CWP (R^2^ = 0.88, Q^2^ = 0.81, CV-ANOVA *p*-value: <0.001; one predictive component). The important significant proteins (including proteoforms), mainly from plasma, are shown in Table 2 and Table 3.

In CON, the protein with the strongest correlation with CPT was the muscle protein keratin type II cytoskeletal I, which is involved in mitochondrial function (Table 2). The most important plasma proteins (i.e., highest VIPpred) were proteoforms of alpha-2-macroglublin, apolipoprotein C-III, and ceruloplasmin from plasma (Table 2). Most proteins were acute phase proteins (Table 2). The significant proteins also included complement factors and 2 apolipoprotein-III proteoforms (associated with immunity). Several of the important proteins had proteoforms (i.e., alpha-1-antitrypsin, alpha-2-macroglobulin, ceruloplasmin, and complement factor C4-B and plasminogen) (Table 2). All proteins except one proteoform of alpha-1-antitrypsin correlated positively with CPT.

In CWP, the most important proteins for CPT were plasma proteins: apolipoprotein A-I, clusterin, Ig kappa chain C region, and alpha-2-macroglobulin (Table 3). The important proteins were acute phase proteins, three complement factors, one chemokine (CCL19), one cytokine (IL-7), two immunoglobulin factors, other factors associated with immunity (apolipoprotein A-I, CD244 (part of the Ig superfamily), and vitamin D-binding protein proteoforms) (Table 3). The only significant muscle protein was creatine kinase M-type, which is involved in the glycolytic pathway. Positive correlations with CPT were found in 12 of 20 proteins. Only two proteins and proteoforms were identical when comparing the important regressors for CPT between CON and CWP (i.e., a proteoform of alpha-1-antitrypsin and a proteoform of plasminogen) (Table 2andTable 3). Alpha-2-macroglobulin was important in both regressions, but the proteoforms differed.

#### 3.4.2. HPT

For HPT, significant regressions were obtained in CON (R^2^ = 0.77, Q^2^ = 0.63, CV-ANOVA *p*-value: < 0.001; one predictive component) and in CWP (R^2^ = 0.77, Q^2^ = 0.61, CV-ANOVA *p*-value: 0.003; one predictive component) (Table 4 and Table 5).

In CON, the most important proteins—mainly plasma proteins—in the regression of HPT were one proteoform of serotransferrin, two proteoforms of alpha-2 macroglobulin, and complement C3 alpha chain (Table 4). In detail, acute phase proteins, two complement factors, two immunoglobulin factors, one chemokine (CCL20), one cytokine (CDCP1), as well as other molecules associated with immunity (i.e., beta-2-glycoprotein 1, EN-RAGE, and vitamin D-binding protein) were important (Table 4). The only muscle protein was phosphoglycerate mutase 2, which is involved in the glycolytic pathway. Several of the identified proteins were detected as different proteoforms: three proteoforms of alpha-2-macroglobulin, three of ceruloplasmin, and two of serotransferrin (Table 4).

In CWP the regression of HPT identified the plasma proteins clusterin, IL-10RB, and CSF-1 as most important (Table 5). In detail, three acute phase proteins; three complement factors; three cytokines (CSF-1, Flt3L, and IL-10RB); one neurotrophin (neurotrophin 3, NT-3); two immunoglobulin-related molecules; and other proteins related to inflammation and immunity (apolipoprotein A-I, TNFRSF9, and vitamin D-binding protein) were important plasma proteins (Table 5). Two muscle proteins related to the glycolytic pathway were also significant regressors (i.e., creatine kinase M-type and phosphoglycerate mutase 2). Two proteins—complement component C7 and immunoglobulin light chain—had two proteoforms among the significant proteins (Table 5). When comparing the important proteins for HPT between CON and CWP, some proteins were identical (phosphoglycerate mutase 2), but the proteoforms for alpha-2-macroglobulin and vitamin D-binding protein were different.

### 3.5. Both Proteins and Clinical Variables as Regressors of CPT and HPT

In the next step, the clinical variables were added to the regressions displayed in Table 2, Table 3, Table 4, Table 5. In the CON, these variables were not important compared to the 20 proteins nor in the regression of CPT (i.e., proteins: R^2^ = 0.94, Q^2^ = 0.76 and CV-ANOVA *p*-value: 0.005 vs. proteins and clinical variables: R^2^ = 0.97, Q^2^ = 0.75 and CV-ANOVA *p*-value: 0.004) nor in the regression of HPT (proteins: R^2^ = 0.77, Q^2^ = 0.63 and CV-ANOVA *p*-value: <0.001 vs. proteins and clinical variables: R^2^ = 0.78, Q^2^ = 0.62 and CV-ANOVA *p*-value: <0.001).

Some of the clinical variables were important for the regressions in CWP. In the regression of CPT, HADS was the 15th most important variable, whereas the other psychometric variables had little importance (proteins: R^2^ = 0.88, Q^2^ = 0.81 and CV-ANOVA *p*-value: <0.001 vs. proteins and clinical variables: R^2^ = 0.95, Q^2^ = 0.80 and CV-ANOVA *p*-value: 0.002). In the regression of HPT, HADS was the most important and QOL the fifth most important regressors. The explained variation increased when the clinical variables were added (proteins: R^2^ = 0.77, Q^2^ = 0.61 and CV-ANOVA *p*-value: 0.003 vs. proteins and clinical variables: R^2^ = 0.99, Q^2^ = 0.79 and CV-ANOVA *p*-value: 0.048). However, HADS was not markedly more important than the most important protein (HADS: VIPpred = 1.42 vs. IL-10rb: VIPpred = 1.40).

### 3.6. Network Analyses

#### 3.6.1. CPT

**Figure 2:**
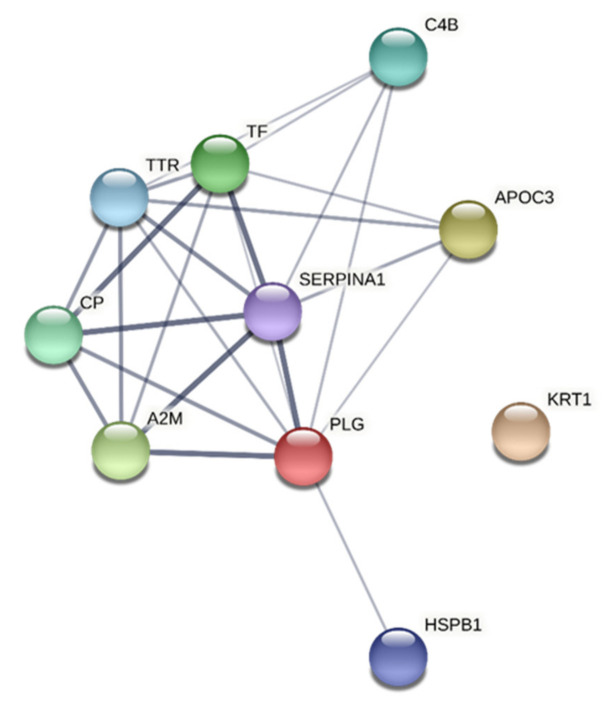
Network analyses of important proteins for CPT in CON. The network had the following characteristics: number of nodes: 10; number of edges: 24; average node degree: 4.8; avg. local clustering coefficient: 0.786; expected number of edges: 1; PPI enrichment *p*-value: <1.0 × 10^−16^. PLG = plasminogen; KRT1 = keratin, type II cytoskeletal 1; APOC3 = apolipoprotein C-III; A2M = alpha-2-macroglobulin; TF = serotransferrin; CP = ceruloplasmin; C4B = complement C4-B; TTR = transthyretin; HSPB1 = heat shock protein beta-1; SERPINA1 = alpha-1-antitrypsin; CON = healthy control group; CPT = cold pain threshold.

The network and enrichment analysis of the important proteins in CON (Table 2) identified a protein–protein interaction network that was significantly enriched (Figure 2). Extracellular region and aspects of secretory and platelet granule had the lowest FDR according to CC (Table 6). MF terms with the lowest FDR were enzyme inhibitor activity and protein binding. The significant BP terms were platelet degranulation, transport aspects, regulated exocytosis, protein activation cascade, and protein metabolic process (Table 6).

**Figure 3:**
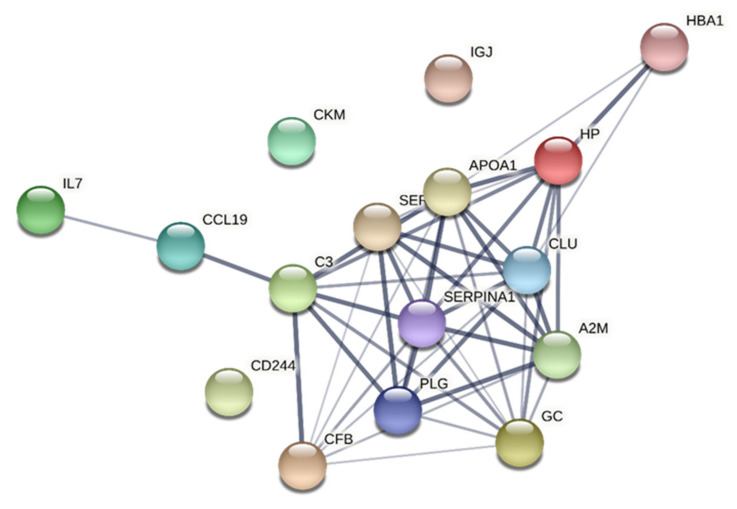
Network analyses of important proteins for CPT in CWP. The network had the following characteristics: number of nodes: 16; number of edges: 45; average node degree: 5.62; avg. local clustering coefficient: 0.636; expected number of edges: 3; PPI enrichment *p*-value: <1.0 × 10^−16^. Note that Ig kappa chain C region is not included in STRING. HP = haptoglobin; CFB = complement factor B; GC = vitamin D-binding protein; C3 = complement C3; IL7 = interleukin-7; CKM = creatine kinase M-type; CCL19 = chemokine (C-C motif) ligand 19; CLU = clusterin; PLG = plasminogen; SERPINA1 = alpha-1-antitrypsin; HBA1 = haemoglobin subunit alpha; IGJ = immunoglobulin J chain; SERPINF2 = alpha-2-antiplasmin; APOA1 = apolipoprotein A-I; CD244 = cluster of differentiation 244, natural killer cell receptor 2B4; A2M = alpha-2-macroglobulin; CWP = chronic widespread pain group; CPT = cold pain threshold.

In CWP, a significantly enriched protein–protein interaction network was identified (Table 7 and Figure 3). CC terms with lowest FDR were related to the extracellular regions and space as well as the lumens of vesicle secretory granule and platelets. MF terms with lowest FDR were signalling receptor and protein binding as well as enzyme and endopeptidase inhibitor activity. Most of the GO terms concerned BP; the most important were platelet degranulation and terms related to the immune system, response to stress and defence, protein activation/cascade, and cytokine regulations (Table 7). Fewer CC, MF, and BP terms were obtained in CON than in CWP (Table 6 and Table 7). Most terms obtained in CON within each of the three areas of GO were also found in CWP.

#### 3.6.2. HPT

**Figure 4:**
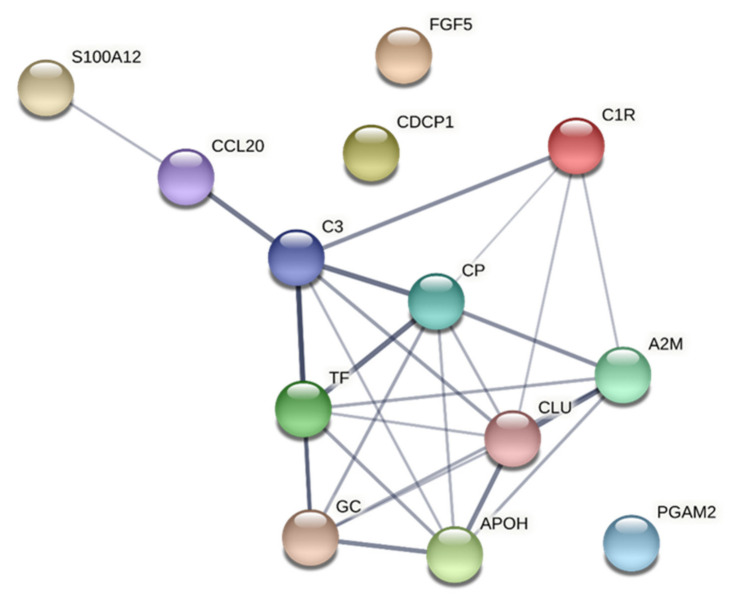
Network analyses of important proteins for HPT in CON. The network had the following characteristics: number of nodes: 13; number of edges: 26; average node degree: 4; avg. local clustering coefficient: 0.586; expected number of edges: 2; PPI enrichment *p*-value: <1.0 × 10^−16^. Note that Ig alpha-2 chain C region and Ig kappa chain C region are not included in STRING. A2M = alpha-2-macroglobulin; APOH = beta-2-glycoprotein 1; CCL20 = chemokine (C-C motif) ligand 20; CDCP1 = CUB domain-containing protein 1; CP = ceruloplasmin; CLU = clusterin; C1R = complement C1r subcomponent; C3 = complement C3; S100A12 = protein S100-A12/EN-RAGE; FGF5 = fibroblast growth factor 5; TF = serotransferrin; PGAM2 = phosphoglycerate mutase 2; GC = vitamin D-binding protein; CON = healthy control group; HPT = heat pain threshold.

**Figure 5:**
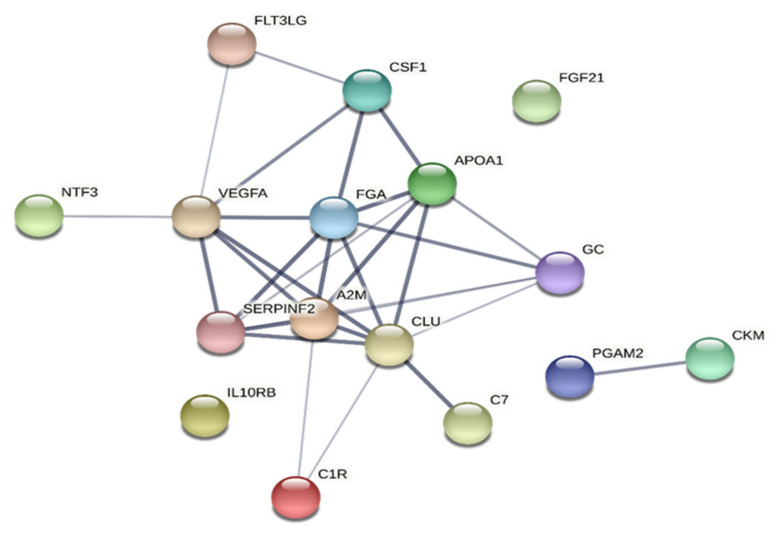
Network analyses of important proteins for HPT in CWP. The network had the following characteristics: number of nodes: 16; number of edges: 29; average node degree: 3.62; avg. local clustering coefficient: 0.71; expected number of edges: 3; PPI enrichment *p*-value: < 1.0 × 10^−16^. Note that Ig light chain is not included in STRING. SERPINF2 = alpha-2-antiplasmin; A2M = alpha-2-macroglobulin; APOA1 = apolipoprotein A-I; CKM = creatine kinase M-type; PGAM2 = phosphoglycerate mutase 2; CLU = clusterin; C1R = complement C1r subcomponent; C7 = complement component C7; CSF1 = colony stimulating factor 1; FGF21 = fibroblast growth factor 21; FGA = fibrinogen alpha chain; FLT3LG = Fms-related tyrosine kinase 3 ligand; IL10RB = interleukin-10 receptor subunit beta; NTF3 = neurotrophin-3; TNFRSF9 = tumour necrosis factor receptor superfamily member 9; VEGFA = vascular endothelial growth factor A; GC = vitamin D-binding protein; CWP = chronic widespread pain group; HPT = heat pain threshold.

Significantly enriched protein–protein interaction networks were found for the proteins strongly associated with HPT both in CON (Table 8 and Figure 4) and in CWP (Table 9 and Figure 5). In CON, CC terms with lowest FDR were extracellular areas and secretory granule lumen (Table 8). An MF term with relatively low FDR was the signalling receptor binding. BP terms with low FDR were aspects of protein activation and complement activation, regulation and response of inflammatory and immune system (Table 8).

In CWP, the CC terms with lowest FDR were associated with extracellular areas, lumens of platelets, and secretory granules (Table 9). The MF terms were related to receptor-related activities and growth factor activity. Most of the GO terms were related to BP (i.e., platelet degranulation, protein activation cascade, protein regulation, complement activation, intracellular signal transduction, and inflammatory response). In addition, response to external stimuli had very low FDR (Table 9). More CC, MF, and BP terms were obtained in CWP than in CON. For example, the number of BP terms with FDR < 0.001 for HPT were 11 in CON and 45 in CWP. Most terms obtained in CON within each of the three areas of GO were also found in CWP.

## 4. Discussion

The three hypotheses stated were conformed and therefore the following major results were noted:

- Patients with CWP had lowered pain thresholds for thermal stimulus; these levels were generally not related to the included clinical variables except for HPT in CWP.
- Patterns of highly interacting proteins mainly from plasma showed strong associations with CPT and HPT both in CWP and in CON.
- Differences in the important proteins for the two thermal pain thresholds were noted between CWP and CON; more complex patterns emerged in CWP.

CWP had lowered pain thresholds for cold and heat (Table 1), a finding that agrees with other studies [54,55,56,57,58]. For both the CWP and CON, CPT and HPT were generally not associated with clinical variables, which agrees with a study of FM [58]. The exception was in CWP: HADS and QOL showed significant associations with HPT, and HADS was a strong regressor of HPT although it was not markedly stronger than the most important proteins. Whiplash associated disorders and chronic pelvic pain were reported to have significant associations between thermal pain thresholds and clinical variables [37,38,59]. The resting-state brain connectome could predict pain thresholds for heat and pressure in healthy subjects [60]. Thus, larger studies need to be conducted to determine the importance of common clinical variables in relation to peripheral and central biomarkers of pain thresholds.

A broad sampling of plasma proteins was applied—i.e., proteins at nano and micro molar concentrations (proteomics) and at picomolar concentrations (cytokines, chemokines and growth factors)—to focus on protein interactions and possible biological processes. The latter approach was advocated before it will be possible to focus on key proteins [36,61] and is increasingly applied in proteomic blood and cerebrospinal fluid studies of CWP and FM [14,23,24,25,26,62]. Plasma/serum proteome studies of CWP and FM cohorts have focussed on differentiating patients from controls, and low-grade peripheral inflammation appears to be involved in pathogenesis and maintenance of CWP and FM [10,17,24,25,26,63]. It is also important to examine the peripheral protein patterns in relation to clinical variables since it cannot be assumed that the same proteins responsible for group differentiation are important for clinical variables [36]. Plasma proteomic studies of CWP and FM cohorts, including the present CWP/FM subjects, report that the important proteins largely differ across clinical variables [10,25,28,34]. As with PPT [34], the important proteins for both thermal pain thresholds differed between CON and CWP (Table 2, Table 3, Table 4, Table 5). When the same protein was identified, the proteoforms generally differed. The most important proteins explained a large proportion of the variations in CPT (88–94%) and in HPT (both 77%) in the two groups (Table 2, Table 3, Table 4, Table 5). These peripheral protein patterns reported in Table 2, Table 3, Table 4, Table 5 reflect tonic/habitual peripheral situations. With the present design, it is not possible to determine which proteins are specifically involved in the direct activation of the different receptors/channels of the nociceptors. If altered peripheral conditions such as inflammation or tissue injury are present peripherally in CWP, it is reasonable to expect sensitized and primed nociceptors [64]. In agreement with this, we report lowered pain thresholds as well as another pattern of peripheral proteins associated with the thermal pain thresholds in CWP.

Most of the proteins reported in Table 2, Table 3, Table 4, Table 5 were associated with either pain, nociception, or immune system, or in combination. Text S2 presents the results of brief literature reviews with special emphasis on earlier proteomic- and inflammation-related studies in relation to nociception and pain conditions. Although the exact proteins differed between the two groups, it was obvious that mostly plasma proteins related to the host defence/immunity—e.g., acute phase proteins, complement factors, immunoglobulin factors, and cytokines/chemokines—were important for the thermal pain thresholds. For example, in CWP, lower pain thresholds for cold (hyperalgesia) were associated with higher levels of most acute phase proteins, most complement factors, two cytokines/chemokines, and a muscle protein (creatine-kinase M-type), and low levels of proteins were associated with anti-inflammation/enhancement of immunity. Thus, in CWP, proteins representing different parts of the immune system were involved, including cytokine/chemokine, whereas in CON, acute phase proteins and their proteoforms dominated. Moreover, heat hyperalgesia (low pain thresholds for heat) in CWP was associated with low levels of two of three acute phase proteins, most complement factors, two immunoglobulin factors, high levels of four cytokines (CSF-1, IL-10Rb, FGF-21, and Flt3l), and neuroprotective factors (NT3 and VEGF-A). Thus, in CWP, more cytokines and fewer acute phase proteins were involved with respect to HPT compared to CON.

Is it reasonable to conclude that plasma proteins of the immune system are significant regressors of thermal pain thresholds? The circulatory system interacts with other tissues and is vital for the immune-system-based host defence mechanisms and tissue homeostasis [65,66]. Nociceptors in the skin are involved both in neuro-immune and neural-vascular interactions [67]. A network of immune cells is found in the skin [68] and immune cells are also recruited from the blood vascular systems [69]. Structural cells of the skin (epithelial cells, endothelial cells, fibroblasts, etc.) express immune regulators and cytokine signalling and contribute to immunity [70,71]. Dys-regulation in the bidirectional signalling between the nociceptive and immune systems may be associated with excitation [72]. The nociceptors are in direct association or near association with nociceptive Schwann cells in the epidermis and form a mesh-like network [73,74]. The intimate bidirectional relationships between the immune system and the nociceptors peripherally may indicate priming of the immune system [75]. The identified proteins correlating with thermal pain thresholds are consistent with the results from plasma/serum studies differentiating CWP vs. CON and FM vs. CON [10,24,25,26]. In addition, acute phase proteins (both positive and negative), complement factors, immunoglobulin factors, and coagulation factors were important. Inflammation is the biological defensive response of the immune system [76,77]. The crosstalk among immunity (innate and adaptive), coagulative/fibrinolytic pathways, and the nervous system is necessary for adequate inflammatory cascade [77]. We found that peripheral cytokines and chemokines are important, which is consistent with results from Bäckryd et al. [63]. The literature seems to focus on the cytokines/chemokines and their roles; indeed, these are important for pain thresholds in CWP. Our results indicate, however, that other parts of the immune system are also important for thermal pain thresholds. The classification of cytokines and chemokines as pro- or anti-inflammatory is not unproblematic since the properties may depend on the microenvironment [78]. The identified muscle proteins (creatine kinase M-type and phosphoglycerate mutase 2) in the regressions concerning CWP agree with our and other studies reporting muscle metabolic and mitochondrial disturbances in FM (a subgroup of CWP) [79,80,81]. Larger studies are needed to confirm these patterns of proteins and in detail elucidate the interactions between the different parts of the immune system and related interacting factors.

Both variables and groups of subjects revealed significantly enriched protein–protein interaction networks (Figure 2, Figure 3, Figure 4, Figure 5). Hence, the groups of plasma proteins generally interact, which is reasonable from a host defence perspective. Different proteoforms were important and sometimes had different signs within a regression (e.g., alpha-1 antitrypsin in the regression of CPT in CON and vitamin D-binding protein in the regression of CPT in CWP). Fewer GO terms were obtained in CON than in CWP. This finding suggests a more complex molecular relationships in CWP and therefore reflects peripheral molecular alterations associated with hyperalgesia in CWP. However, because STRING cannot handle different proteoforms, fewer proteins are included in analyses of CON than for CWP. In future larger studies, it will be important to scrutinize in more detail the pattern of proteoforms and how they may be altered in CWP.

The primary origin and maintenance factors for the pathophysiological alterations in CWP and in the FM subgroup are not known. Morphological and functional alterations both in the CNS (e.g., neuroinflammation, opioidergic dysregulation, and central sensitization) and in the periphery (e.g., systemic low-grade inflammation, small fibre impairment, reduced skin innervation, and muscle alterations such as mitochondrial disturbance) are found [77,82,83,84,85,86,87,88,89,90,91,92,93]. These mechanisms may be present simultaneously, indicating complicated interactions between peripheral and central processes in CWP including FM that contribute to pain and hypersensitivity. The present study (mainly consisting of FM patients), other proteomic and large cytokine/chemokine studies, and other peripheral studies may challenge the IASP’s definition that FM is a nociplastic condition [29].

### Strengths and Limitations

An important strength of the study is that it examines a deficiently studied area in CWP including FM, i.e., whether peripheral molecular changes are associated with thermal pain thresholds. The cross-sectional design and the small sample size are limitations of this exploratory pilot study. Larger studies with repeated measures and population-based studies are important for validation [94]. As suggested in a previous systematic review [36], we applied MVDA for relating clinical parameters (i.e., pain thresholds) to many possibly intercorrelated proteins. The removal procedure of, e.g., albumin and IgG (i.e., large abundant proteins), could have removed important low abundant proteins. Proteoforms of a protein and post translational modifications can be detected by 2-DE, which is important as several of the significant proteins were expressed as different proteoforms, findings also reported earlier [10,28,34]. The ACR 1990 classification criteria for CWP and FM was used to simplify comparisons with earlier studies. In future studies, both ACR 1990 and the newer 2016 criteria should be used to optimize comparisons with other studies.

## 5. Conclusions and Clinical Implications

The present study contributes towards an emerging picture that peripheral proteins are associated with pain thresholds in CWP and FM; we have earlier reported such associations for pressure pain thresholds [25,34]. Hence, patterns of highly interacting proteins mainly from plasma showed strong associations with CPT and HPT both in CWP and in CON. Although different proteins were important in the two groups, there were also similarities; proteins related to the host defence/immunity such as acute phase proteins, complement factors, immunoglobulin factors, and cytokines/chemokines (not in CON for CPT) were important habitual/tonic factors for thermal pain thresholds. Although peripheral proteins contribute to thermal pain thresholds, central factors and complex interactions between peripheral and central factors may contribute to pain thresholds in CWP. The present study indicates that peripheral molecular factors are important for the clinical presentations in CWP and future larger studies may contribute to an increased understanding of the molecular mechanisms involved in CWP including FM, which in turn may contribute to the development of new therapies.

## Figures and Tables

**Table 1:** Background and PROM data (mean and SD) together with CPT and HPT over trapezius (mean of right and left sides and SD) for CON and CWP groups. Most of the background and PROM data were published elsewhere [28,34].

Group	CON	*n* = 23	CWP	*n* = 15	Statistics
Variables	Mean	SD	Mean	SD	*p*-Value
Age (y)	41.0	10.2	49.2	8.9	0.014
BMI (kg/m^2^)	24.0	2.8	26.0	5.0	0.185
NRS-neck	0.0	0.0	5.7	2.4	<0.001
NRS-shoulders	0.0	0.0	5.7	1.9	<0.001
NRS-whole body	0.0	0.0	4.9	2.0	<0.001
HADS	3.3	2.8	14.0	5.3	<0.001
PCS	6.4	6.4	13.0	7.5	0.010
QOLS	93.1	9.7	82.5	13.1	0.013
CPT (°C)	11.6	4.0	15.8	5.7	0.020
HPT (°C)	48.0	1.4	46.6	2.0	0.027

Notes: CON = healthy control group; CWP = chronic widespread pain group; BMI = body mass index; NRS = numeric rating scale for pain intensity; HADS = Hospital Anxiety and Depression Scale; PCS = Pain Catastrophizing Scale; QOLS = Quality of Life Scale; CPT = cold pain threshold; HPT = heat pain threshold. PROM = patient reported outcome measures.

**Table 2:** OPLS regression of CPT in CON; significant proteins (in alphabetical order) are presented according to VIPpred. Characterization is based upon brief reviews presented in Text S2. Proteins in italics are from muscle biopsy and the others are plasma proteins. A positive sign of p(corr) indicates that a high level of the protein is associated with a low pain threshold for cold.

Spot No.	Accession No.	Protein	VIPpred	p(corr)	Characterization
3420	P01009	Alpha-1-antitrypsin	2.16	−0.45	Acute phase protein
3712	P01009	Alpha-1-antitrypsin	2.14	0.44	Acute phase protein
5811	P01023	Alpha-2-macroglobulin	3.12	0.64	Acute phase protein
4902	P01023	Alpha-2-macroglobulin	2.19	0.45	Acute phase protein
1055	P02656	Apolipoprotein C-III	3.42	0.73	Antiinflammation and enhance immunity
1054	P02656	Apolipoprotein C-III	2.15	0.46	Antiinflammation and enhance immunity
4814	P00450	Ceruloplasmin	3.20	0.67	Acute phase protein
3817	P00450	Ceruloplasmin	2.78	0.56	Acute phase protein
3904	P00450	Ceruloplasmin	2.32	0.47	Acute phase protein
4832	P00450	Ceruloplasmin	2.20	0.43	Acute phase protein
4816	P00450	Ceruloplasmin	2.19	0.44	Acute phase protein
7216	P0C0L5	Complement C4-B	2.95	0.60	Complement factors
8101	P0C0L5	Complement C4-B	2.57	0.53	Complement factors
*B3540*	*P04792*	*Heat shock protein beta-1*	2.59	0.63	Protective
*B1834*	*P04264*	*Keratin, type II cytoskeletal I*	2.99	0.82	Mitochondrial function
8901	P00747	Plasminogen	2.53	0.52	Acute phase protein
7820	P00747	Plasminogen	2.51	0.52	Acute phase protein
7819	P00747	Plasminogen	2.42	0.50	Acute phase protein
7702	P02787	Serotransferrin	2.29	0.50	Acute phase protein
1003	P02766	Transthyretin	2.38	0.49	Acute phase protein
		R^2^ = 0.94			
		Q^2^ = 0.76			
		CV-ANOVA *p* = 0.0047			

VIPpred and p(corr) are reported for each regressor, i.e., the loading of each variable scaled as a correlation coefficient and therefore standardizing the range from −1 to +1. A variable/regressor was considered significant when VIPpred > 1.0 and absolute p(corr) ≥ 0.40. The sign of p(corr) indicates the direction of the correlation with the dependent variable (+ = positive correlation; − = negative correlation). CON = healthy control group; CPT = cold pain threshold. Spot no. refers to identified protein spots in previous publications. For visual location of proteins on 2-DE gel and comparisons of proteoforms see [10,23,27,28].

**Table 3:** OPLS regression of CPT in CWP; significant proteins (in alphabetical order) are presented according to VIPpred. Characterization is based on brief reviews presented in Text S2. Proteins in italics are muscle proteins and the others are plasma proteins. A positive sign of p(corr) indicates that a high level of the protein is associated with a low pain threshold for cold (hyperalgesia).

Spot No.	Accession No.	Protein	VIPpred	p(corr)	Characterization
3712	P01009	Alpha-1-antitrypsin	2.17	0.58	Acute phase protein +
5906	P01023	Alpha-2-macroglobulin	2.62	0.70	Acute phase protein +
4608	P08697	Alpha-2-antiplasmin	2.10	0.56	Acute phase protein +
3606	P08697	Alpha-2-antiplasmin	1.98	0.53	Acute phase protein +
1102	P02647	Apolipoprotein A-I	2.81	−0.75	Antiinflammation
NA	Q99731	CCL19	2.32	0.66	Chemokine
NA	Q9BZW8	CD244	2.23	0.63	Immunity, Ig superfamily
3214	P10909	Clusterin	2.73	−0.73	Neuroprotective, etc.
7901	P00751	Complement factor B	2.48	0.66	Complement factors
6841	P01024	Complement C3c alpha chain	1.98	0.53	Complement factors
145	P01024	Complement C3c alpha chain fragment 2	1.92	−0.52	Complement factors
*B5736*	*P06732*	*Creatine kinase M-type*	2.46	0.66	ATP production
3105	P00738	Haptoglobin	2.06	−0.55	Acute phase protein +
9009	P69905	Haemoglobin subunit alpha	1.98	−0.53	Blood—haemoglobin +
9001	P01834	Ig kappa chain C region	2.63	−0.71	Immunoglobulin
166	P01591	Ig J chain	2.59	−0.69	Immunoglobulin
NA	P13232	IL-7	1.97	0.56	Cytokine
8901	P00747	Plasminogen	1.99	0.53	Acute phase protein +
3408	P02774	Vitamin D-binding protein	2.43	−0.65	Immunity, etc.
2502	P02774	Vitamin D-binding protein	2.05	0.55	Immunity, etc.
		R^2^ = 0.88			
		Q^2^ = 0.81			
		CV-ANOVA *p* < 0.001			

VIPpred and p(corr) are reported for each regressor, i.e., the loading of each variable scaled as a correlation coefficient and therefore standardizing the range from −1 to +1. A variable/regressor was considered significant when VIPpred > 1.0 and absolute p(corr) ≥ 0.40. The sign of p(corr) indicates the direction of the correlation with the dependent variable (+ = positive correlation; − = negative correlation). CCL19 = chemokine (C-C motif) ligand 19; CD244 = cluster of differentiation 244; IL-7 = interleukin-7; Ig = immunoglobulin; CWP = chronic widespread pain group; CPT = cold pain threshold. Spot no. refers to identified protein spots in previous publications. For visual location of proteins on 2-DE gel and comparisons of proteoforms see [10,23,27,28].

**Table 4:** OPLS regression of HPT in CON; significant proteins (in alphabetical order) are presented according to VIPpred. Characterization is based on brief reviews (Text S2). Proteins in italics are muscle proteins and the others are plasma proteins. A negative sign for p(corr) indicates that high level of the protein is associated with a low pain threshold for heat.

Spot No.	Accession No.	Protein	VIPpred	p(corr)	Characterization
5811	P01023	Alpha-2-macroglobulin	2.77	−0.59	Acute phase protein
4902	P01023	Alpha-2-macroglobulin	2.65	−0.58	Acute phase protein
5821	P01023	Alpha-2-macroglobulin	2.01	−0.43	Acute phase protein
6528	P02749	Beta-2-glycoprotein 1	2.40	0.51	Immunity, regulation of complement/coagulation
NA	P78556	CCL20	2.05	−0.48	Chemokine
NA	Q9H5V8	CDCP1	2.11	0.50	Cytokine
4830	P00450	Ceruloplasmin	2.59	−0.53	Acute phase protein
3817	P00450	Ceruloplasmin	2.45	−0.51	Acute phase protein
4832	P00450	Ceruloplasmin	2.13	−0.44	Acute phase protein
1105	P10909	Clusterin	2.21	−0.46	Neuroprotective, etc.
4914	P00736	Complement C1r subcomponent	2.15	−0.44	Complement factors
6842	P01024	Complement C3 alpha chain	2.62	0.55	Complement factors
NA	P80511	EN-RAGE	2.04	0.48	Immunity, etc.
NA	P12034	FGF-5	2.09	0.48	Tissue repair and regulator Schwann cells
5511	P01877	Ig alpha-2 chain C region	2.28	0.46	Immunoglobulin
8001	P01834	Ig kappa chain C region	2.05	−0.43	Immunoglobulin
*B7525*	*P15259*	*Phosphoglycerate mutase 2*	*2.02*	−*0.51*	Glycolytic pathway
7744	P02787	Serotransferrin	2.51	0.54	Acute phase protein
7731	P02787	Serotransferrin	2.84	0.61	Acute phase protein
3408	P02774	Vitamin D-binding protein	2.19	−0.47	Immunity, etc.
		R^2^ = 0.77			
		Q^2^ = 0.63			
		CV-ANOVA *p* < 0.001			

VIPpred and p(corr) are reported for each regressor, i.e., the loading of each variable scaled as a correlation coefficient and therefore standardizing the range from −1 to +1. A variable/regressor was considered significant when VIPpred > 1.0 and absolute p(corr) ≥ 0.40. The sign of p(corr) indicates the direction of the correlation with the dependent variable (+ = positive correlation; − = negative correlation). CCL20 = chemokine (C-C motif) ligand 20; CDCP1 = CUB-domain containing protein 1; EN-RAGE = protein S100-A12; FGF-5 = fibroblast growth factor 5; Ig = immunoglobulin; CON = healthy control group; HPT = heat pain threshold. Spot no. refers to identified protein spots in previous publications. For visual location of proteins on 2-DE gel and comparisons of proteoforms see [10,23,27,28].

**Table 5:** OPLS regression of HPT in CWP; significant proteins (in alphabetical order) are presented according to VIPpred. Characterization is based on brief reviews (Text S2). Proteins in italics are muscle proteins and the others are plasma proteins. A negative sign for p(corr) indicates that high level of the protein is associated with a low pain threshold for heat (hyperalgesia).

Spot No.	Accession No.	Protein	VIPpred	p(corr)	Characterization
3619	P08697	Alpha-2-antiplasmin	2.02	0.51	Acute phase protein
6903	P01023	Alpha-2-macroglobulin	2.02	0.51	Acute phase protein
1102	P02647	Apolipoprotein A-I	2.00	0.51	Antiinflammation
3214	P10909	Clusterin	2.71	0.69	Neuroprotective, etc.
5819	P00736	Complement C1r subcomponent	1.96	−0.50	Complement factors
6844	P10643	Complement component C7	2.33	0.59	Complement factors
6845	P10643	Complement component C7	1.97	0.50	Complement factors
*B5736*	*P06732*	*Creatine kinase M-type*	2.09	−0.53	ATP production
NA	P09603	CSF-1	2.48	−0.66	Cytokine
NA	Q9NSA1	FGF-21	2.02	−0.54	Cytokine
8621	P02671	Fibrinogen alpha chain	2.35	−0.60	Acute phase protein
NA	P49771	FIt3L	2.35	−0.62	Cytokine
9006	Q0KKI6	Ig light chain	2.15	0.55	Immunoglobulin
9008	Q0KKI6	Ig light chain	2.18	0.56	Immunoglobulin
NA	Q08334	IL-10RB	2.56	−0.68	Cytokine
NA	P20783	Neurotrophin 3	1.97	−0.52	Neurons- repair and growth
*B7523*	*P15259*	*Phosphoglycerate mutase 2*	1.97	0.50	Glycolytic pathway
NA	Q07011	TNFRSF9	2.02	−0.53	Inflammation development
NA	P15692	VEGF-A	1.99	−0.53	Neuroprotective and pro-nociceptive
2502	P02774	Vitamin D-binding protein	1.97	−0.50	Immunity, etc.
		R^2^ = 0.77			
		Q^2^ = 0.61			
		CV-ANOVA *p* = 0.0033			

VIPpred and p(corr) are reported for each regressor, i.e., the loading of each variable scaled as a correlation coefficient and therefore standardizing the range from −1 to +1. A variable/regressor was considered significant when VIP > 1.0 and absolute p(corr) ≥ 0.40. The sign of p(corr) indicates the direction of the correlation with the dependent variable (+ = positive correlation; − = negative correlation). CSF-1 = colony stimulating factor 1; FGF-21 = fibroblast growth factor 21; FIt3L = FMS-like tyrosine kinase 3 ligand; IL-10RB = interleukin 10 receptor, beta subunit: TNFRSF9 = tumour necrosis factor receptor superfamily member 9: VEGF-A = vascular endothelial growth factor A; Ig = immunoglobulin; CWP = chronic widespread pain group; HPT = heat pain threshold. Spot no. refers to identified protein spots in previous publications. For visual location of proteins on 2-DE gel and comparisons of proteoforms see [10,23,27,28].

**Table 6:** The most significant GO terms within cellular component (CC), molecular function (MF), and biological process (BP) for CPT in CON.

Category.	Term ID	Term Description	Gene Count	Strength	FDR	Matching Proteins in Network
CC	GO:0005576	extracellular region	9	0.85	1.08 × 10^−5^	APOC3, TTR, KRT1, CP, PLG, A2M, TF, C4B, SERPINA1
CC	GO:0034774	secretory granule lumen	5	1.48	1.96 × 10^−5^	TTR, PLG, A2M, TF, SERPINA1
CC	GO:0030141	secretory granule	6	1.15	2.77 × 10^−5^	TTR, KRT1, PLG, A2M, TF, SERPINA1
CC	GO:0031093	platelet alpha granule lumen	3	1.94	0.00010	PLG, A2M, SERPINA1
CC	GO:0031232	extrinsic component of external side of plasma membrane	2	2.75	0.00014	PLG, TF
CC	GO:0031410	cytoplasmic vesicle	7	0.79	0.00028	APOC3, TTR, KRT1, PLG, A2M, TF, SERPINA1
MF	GO:0004857	enzyme inhibitor activity	5	1.4	7.89 × 10^−5^	APOC3, HSPB1, A2M, C4B, SERPINA1
MF	GO:0005515	protein binding	10	0.47	0.0010	APOC3, TTR, HSPB1, KRT1, CP, PLG, A2M, TF, C4B, SERPINA1
BP	GO:0002576	platelet degranulation	4	1.78	0.00011	PLG, A2M, TF, SERPINA1
BP	GO:0006810	transport	10	0.68	0.00011	APOC3, TTR, HSPB1, KRT1, CP, PLG, A2M, TF, C4B, SERPINA1
BP	GO:0045055	regulated exocytosis	6	1.23	0.00011	TTR, KRT1, PLG, A2M, TF, SERPINA1
BP	GO:0065008	regulation of biological quality	9	0.69	0.00018	APOC3, TTR, HSPB1, KRT1, CP, PLG, A2M, TF, SERPINA1
BP	GO:0016192	vesicle-mediated transport	7	0.91	0.00024	TTR, KRT1, PLG, A2M, TF, C4B, SERPINA1
BP	GO:0072376	protein activation cascade	3	1.9	0.00037	KRT1, A2M, C4B
BP	GO:0019538	protein metabolic process	9	0.62	0.00038	APOC3, TTR, KRT1, CP, PLG, A2M, TF, C4B, SERPINA1
BP	GO:0043062	extracellular structure organization	4	1.36	0.00082	APOC3, TTR, PLG, A2M

Gene count = observed gene count; FDR = false discovery rate; PLG = plasminogen; KRT1 = keratin, type II cytoskeletal 1; APOC3 = apolipoprotein C-III; A2M = alpha-2-macroglobulin; TF = serotransferrin; CP = ceruloplasmin; C4B = complement C4-B; TTR = transthyretin; HSPB1 = heat shock protein beta-1; SERPINA1 = alpha-1-antitrypsin; CON = healthy control group; CPT = cold pain threshold.

**Table 7:** The most significant GO terms within cellular component (CC), molecular function (MF), and biological process (BP) for CPT in CWP.

Category	Term ID	Term Description	Gene Count	Strength	FDR	Matching Proteins in Network
CC	GO:0060205	cytoplasmic vesicle lumen	9	1.51	1.88 × 10^−10^	APOA1, C3, PLG, CLU, SERPINF2, HBA1, A2M, HP, SERPINA1
CC	GO:0005576	extracellular region	14	0.83	1.14 × 10^−9^	APOA1, C3, IGJ, IL7, CCL19, PLG, CLU, SERPINF2, HBA1, A2M, HP, SERPINA1, CFB, GC
CC	GO:0005615	extracellular space	11	1.07	1.96 × 10^−9^	APOA1, C3, IGJ, IL7, CCL19, PLG, CLU, SERPINF2, HBA1, HP, SERPINA1
CC	GO:0034774	secretory granule lumen	8	1.48	1.96 × 10^−9^	APOA1, C3, PLG, CLU, SERPINF2, A2M, HP, SERPINA1
CC	GO:0031093	platelet alpha granule lumen	5	1.95	4.95 × 10^−8^	PLG, CLU, SERPINF2, A2M, SERPINA1
CC	GO:0034366	spherical high-density lipoprotein particle	3	2.61	1.37 × 10^−6^	APOA1, CLU, HP
CC	GO:0071682	endocytic vesicle lumen	3	2.29	6.95 × 10^−6^	APOA1, HBA1, HP
CC	GO:0031838	haptoglobin-haemoglobin complex	2	2.79	6.56 × 10^−5^	HBA1, HP
CC	GO:0009986	cell surface	5	0.95	0.00098	APOA1, PLG, CLU, SERPINF2, CD244
MF	GO:0005102	signalling receptor binding	9	0.86	9.94 × 10^−5^	APOA1, C3, IGJ, IL7, CCL19, PLG, CLU, A2M, CD244
MF	GO:0004857	enzyme inhibitor activity	5	1.2	0.00070	APOA1, C3, SERPINF2, A2M, SERPINA1
MF	GO:0004866	endopeptidase inhibitor activity	4	1.46	0.00070	C3, SERPINF2, A2M, SERPINA1
MF	GO:0005515	protein binding	14	0.41	0.00070	APOA1, C3, IGJ, IL7, CCL19, PLG, CLU, SERPINF2, A2M, HP, CD244, SERPINA1, CFB, GC
BP	GO:0002576	platelet degranulation	6	1.75	8.75 × 10^−7^	APOA1, PLG, CLU, SERPINF2, A2M, SERPINA1
BP	GO:0002697	regulation of immune effector process	7	1.37	4.74 × 10^−6^	APOA1, C3, CCL19, CLU, A2M, CD244, CFB
BP	GO:0032940	secretion by cell	9	1.06	5.70 × 10^−6^	APOA1, C3, CCL19, PLG, CLU, SERPINF2, A2M, HP, SERPINA1
BP	GO:0006950	response to stress	13	0.69	7.61 × 10^−6^	APOA1, C3, IGJ, CCL19, PLG, CLU, SERPINF2, HBA1, A2M, HP, CD244, SERPINA1, CFB
BP	GO:0006959	humoral immune response	6	1.46	7.61 × 10^−6^	C3, IGJ, IL7, CCL19, CLU, CFB
BP	GO:0045055	regulated exocytosis	8	1.15	7.61 × 10^−6^	APOA1, C3, PLG, CLU, SERPINF2, A2M, HP, SERPINA1
BP	GO:0070613	regulation of protein processing	5	1.72	7.61 × 10^−6^	C3, CLU, SERPINF2, A2M, CFB
BP	GO:0030449	regulation of complement activation	4	1.97	1.16 × 10^−5^	C3, CLU, A2M, CFB
BP	GO:0006952	defence response	9	0.95	1.23 × 10^−5^	C3, IGJ, CCL19, CLU, SERPINF2, HP, CD244, SERPINA1, CFB
BP	GO:0016192	vesicle-mediated transport	10	0.86	1.23 × 10^−5^	APOA1, C3, IGJ, PLG, CLU, SERPINF2, HBA1, A2M, HP, SERPINA1
BP	GO:2000257	regulation of protein activation cascade	4	1.96	1.23 × 10^−5^	C3, CLU, A2M, CFB
BP	GO:0001817	regulation of cytokine production	7	1.14	2.26 × 10^−5^	APOA1, C3, IL7, CCL19, CLU, SERPINF2, CD244
BP	GO:0032101	regulation of response to external stimulus	8	1.01	2.26 × 10^−5^	APOA1, C3, CCL19, PLG, CLU, SERPINF2, A2M, CFB
BP	GO:0072376	protein activation cascade	4	1.82	2.73 × 10^−5^	C3, CLU, A2M, CFB
BP	GO:0001819	positive regulation of cytokine production	6	1.27	2.82 × 10^−5^	C3, IL7, CCL19, CLU, SERPINF2, CD244
BP	GO:0002673	regulation of acute inflammatory response	4	1.73	5.46 × 10^−5^	C3, CLU, A2M, CFB
BP	GO:0006955	immune response	9	0.85	5.46 × 10^−5^	C3, IGJ, IL7, CCL19, CLU, HP, CD244, SERPINA1, CFB
BP	GO:0006954	inflammatory response	6	1.18	8.23 × 10^−5^	C3, CCL19, CLU, SERPINF2, HP, SERPINA1
BP	GO:0043086	negative regulation of catalytic activity	7	1.02	9.16 × 10^−5^	APOA1, C3, IL7, SERPINF2, A2M, HP, SERPINA1
BP	GO:0098542	defence response to other organisms	7	1.0	0.00013	C3, IGJ, CCL19, CLU, HP, CD244, CFB

Gene count = observed gene count; FDR = false discovery rate. Note that for BP is shown the 20 terms with lowest FDR. HP = haptoglobin; CFB = complement factor B; GC = vitamin D-binding protein; C3 = complement C3; IL7 = interleukin-7; CKM = creatine kinase M-type; CCL19 = chemokine (C-C motif) ligand 19; CLU = clusterin; PLG = plasminogen; SERPINA1 = alpha-1-antitrypsin; HBA1 = haemoglobin subunit alpha; IGJ = immunoglobulin J chain; SERPINF2 = alpha-2-antiplasmin; APOA1 = apolipoprotein A-I; CD244 = cluster of differentiation 244, natural killer cell receptor 2B4; A2M = alpha-2-macroglobulin; CWP = chronic widespread pain group; CPT = cold pain threshold.

**Table 8:** The most significant GO terms within cellular component (CC), molecular function (MF), and biological Process (BP) for HPT in CON.

Category	Term ID	Term Description	Gene Count	Strength	FDR	Matching Proteins in Network
CC	GO:0005576	extracellular region	12	0.86	2.37 × 10^−8^	APOH, C3, CP, CDCP1, FGF5, CLU, A2M, CCL20, S100A12, TF, GC, C1R
CC	GO:0034774	secretory granule lumen	6	1.45	1.74 × 10^−6^	APOH, C3, CLU, A2M, S100A12, TF
CC	GO:0005615	extracellular space	7	0.97	5.86 × 10^−5^	APOH, C3, CP, FGF5, CLU, CCL20, C1R
MF	GO:0005102	signalling receptor binding	7	0.84	0.0025	C3, FGF5, CLU, A2M, CCL20, S100A12, TF
MF	GO:0005507	copper ion binding	2	1.73	0.0435	CP, S100A12
BP	GO:0072376	protein activation cascade	5	2.01	1.05 × 10^−6^	APOH, C3, CLU, A2M, C1R
BP	GO:0030449	regulation of complement activation	4	2.06	1.88 × 10^−5^	C3, CLU, A2M, C1R
BP	GO:2000257	regulation of protein activation cascade	4	2.05	1.88 × 10^−5^	C3, CLU, A2M, C1R
BP	GO:0002673	regulation of acute inflammatory response	4	1.82	6.69 × 10^−5^	C3, CLU, A2M, C1R
BP	GO:0006959	humoral immune response	5	1.48	6.69 × 10^−5^	C3, CLU, CCL20, S100A12, C1R
BP	GO:0070613	regulation of protein processing	4	1.72	0.00012	C3, CLU, A2M, C1R
BP	GO:0002576	platelet degranulation	4	1.67	0.00014	APOH, CLU, A2M, TF
BP	GO:0006958	complement activation, classical pathway	3	2.12	0.00016	C3, CLU, C1R
BP	GO:0050727	regulation of inflammatory response	5	1.35	0.00016	C3, CLU, A2M, S100A12, C1R
BP	GO:0045055	regulated exocytosis	6	1.12	0.00019	APOH, C3, CLU, A2M, S100A12, TF
BP	GO:0032101	regulation of response to external stimulus	6	0.98	0.00087	APOH, C3, CLU, A2M, S100A12, C1R

Gene count = observed gene count; FDR = false discovery rate. Note that for MF none of the displayed terms had FDR < 0.001. A2M = alpha-2-macroglobulin; APOH = beta-2-glycoprotein 1; CCL20 = chemokine (C-C motif) ligand 20; CDCP1 = CUB domain-containing protein 1; CP = ceruloplasmin; CLU = clusterin; C1R = complement C1r subcomponent; C3 = complement C3; S100A12 = protein S100-A12/EN-RAGE; FGF5 = fibroblast growth factor 5; TF = serotransferrin; PGAM2 = phosphoglycerate mutase 2; GC = vitamin D-binding protein; CON = healthy control group; HPT = heat pain threshold.

**Table 9:** The most significant GO terms within cellular component (CC), molecular function (MF), and biological process (BP) for HPT in CWP.

Category	Term ID	Term Description	Gene Count	Strength	FDR	Matching Proteins in Network
CC	GO:0005576	extracellular region	14	0.81	1.36 × 10^−8^	APOA1, FGA, CLU, SERPINF2, C7, A2M, CSF1, NTF3, GC, C1R, FLT3LG, FGF21, VEGFA, TNFRSF9
CC	GO:0031093	platelet alpha granule lumen	5	1.93	1.67 × 10^−7^	FGA, CLU, SERPINF2, A2M, VEGFA
CC	GO:0005615	extracellular space	10	1.01	1.76 × 10^−7^	APOA1, FGA, CLU, SERPINF2, CSF1, C1R, FLT3LG, FGF21, VEGFA, TNFRSF9
CC	GO:0034774	secretory granule lumen	6	1.33	4.05 × 10^−6^	APOA1, FGA, CLU, SERPINF2, A2M, VEGFA
CC	GO:0009986	cell surface	7	1.07	1.11 × 10^−5^	APOA1, FGA, CLU, SERPINF2, FLT3LG, VEGFA, TNFRSF9
CC	GO:0005577	fibrinogen complex	2	2.46	0.00031	FGA, SERPINF2
CC	GO:0034366	spherical high-density lipoprotein particle	2	2.41	0.00035	APOA1, CLU
MF	GO:0005102	signalling receptor binding	9	0.84	0.00020	APOA1, FGA, CLU, A2M, CSF1, NTF3, FLT3LG, FGF21, VEGFA
MF	GO:0048018	receptor ligand activity	6	1.18	0.00020	APOA1, CSF1, NTF3, FLT3LG, FGF21, VEGFA
MF	GO:0008083	growth factor activity	4	1.46	0.00037	CSF1, NTF3, FGF21, VEGFA
BP	GO:0002576	platelet degranulation	6	1.75	7.32 × 10^−7^	APOA1, FGA, CLU, SERPINF2, A2M, VEGFA
BP	GO:0032101	regulation of response to external stimulus	10	1.11	7.32 × 10^−7^	APOA1, FGA, CLU, SERPINF2, C7, A2M, CSF1, NTF3, C1R, VEGFA
BP	GO:2000257	regulation of protein activation cascade	5	2.05	7.32 × 10^−7^	FGA, CLU, C7, A2M, C1R
BP	GO:0072376	protein activation cascade	5	1.92	1.47 × 10^−6^	FGA, CLU, C7, A2M, C1R
BP	GO:0051246	regulation of protein metabolic process	12	0.74	1.02 × 10^−5^	APOA1, FGA, CLU, SERPINF2, C7, A2M, CSF1, NTF3, C1R, FLT3LG, FGF21, VEGFA
BP	GO:0070613	regulation of protein processing	5	1.72	1.02 × 10^−5^	CLU, SERPINF2, C7, A2M, C1R
BP	GO:0019220	regulation of phosphate metabolic process	10	0.87	1.80 × 10^−5^	APOA1, PGAM2, FGA, CLU, SERPINF2, CSF1, NTF3, FLT3LG, FGF21, VEGFA
BP	GO:0030449	regulation of complement activation	4	1.97	1.80 × 10^−5^	CLU, C7, A2M, C1R
BP	GO:0001934	positive regulation of protein phosphorylation	8	1.02	3.63 × 10^−5^	FGA, CLU, SERPINF2, CSF1, NTF3, FLT3LG, FGF21, VEGFA
BP	GO:0001932	regulation of protein phosphorylation	9	0.9	3.74 × 10^−5^	APOA1, FGA, CLU, SERPINF2, CSF1, NTF3, FLT3LG, FGF21, VEGFA
BP	GO:0002682	regulation of immune system process	9	0.9	3.74 × 10^−5^	APOA1, FGA, CLU, C7, A2M, CSF1, C1R, FLT3LG, VEGFA
BP	GO:1902533	positive regulation of intracellular signal transduction	8	1.01	3.74 × 10^−5^	APOA1, FGA, CLU, SERPINF2, CSF1, NTF3, FGF21, VEGFA
BP	GO:0048584	positive regulation of response to stimulus	10	0.77	5.42 × 10^−5^	APOA1, FGA, CLU, SERPINF2, C7, CSF1, NTF3, C1R, FGF21, VEGFA
BP	GO:0002673	regulation of acute inflammatory response	4	1.73	6.68 × 10^−5^	CLU, C7, A2M, C1R
BP	GO:0009605	response to external stimulus	10	0.75	7.61 × 10^−5^	APOA1, IL10RB, FGA, CLU, C7, CSF1, NTF3, C1R, FGF21, VEGFA
BP	GO:0010811	positive regulation of cell-substrate adhesion	4	1.65	0.00011	APOA1, FGA, CSF1, VEGFA
BP	GO:1902531	regulation of intracellular signal transduction	9	0.8	0.00015	APOA1, FGA, CLU, SERPINF2, A2M, CSF1, NTF3, FGF21, VEGFA
BP	GO:0002684	positive regulation of immune system process	7	0.99	0.00017	FGA, CLU, C7, CSF1, C1R, FLT3LG, VEGFA
BP	GO:0006958	complement activation, classical pathway	3	2.03	0.00017	CLU, C7, C1R
BP	GO:0048583	regulation of response to stimulus	12	0.58	0.00017	APOA1, FGA, CLU, SERPINF2, C7, A2M, CSF1, NTF3, C1R, FLT3LG, FGF21, VEGFA

Gene count = observed gene count; FDR = false discovery rate. Note that for BP is shown the 20 terms with lowest FDR. SERPINF2 = alpha-2-antiplasmin; A2M = alpha-2-macroglobulin; APOA1 = apolipoprotein A-I; CKM = creatine kinase M-type; PGAM2 = phosphoglycerate mutase 2; CLU = clusterin; C1R = complement C1r subcomponent; C7 = complement component C7; CSF1 = macrophage colony-stimulating factor 1; FGF21 = fibroblast growth factor 21; FGA = fibrinogen alpha chain; FLT3LG = Fms-related tyrosine kinase 3 ligand; IL10RB = interleukin-10 receptor subunit beta; NTF3 = neurotrophin-3; TNFRSF9 = tumour necrosis factor receptor superfamily member 9; VEGFA = vascular endothelial growth factor A; GC = vitamin D-binding protein; CWP = chronic widespread pain group; HPT = heat pain threshold.

## Data Availability

The datasets generated and analysed in this study are not publicly available as the Ethical Review Board has not approved the public availability of these data.

## Supplementary Materials

The following are available online at https://www.mdpi.com/article/10.3390/jcm10163652/s1, Text S1: Regression of CPT an HPT using clinical variables as regressors, Text S2: Individual proteins.

## References

[1] Mansfield K.E., Sim J., Jordan J.L., Jordan K.P. (2016). A systematic review and meta-analysis of the prevalence of chronic widespread pain in the general population. Pain.

[2] Cimmino M.A., Ferrone C., Cutolo M. (2011). Epidemiology of chronic musculoskeletal pain. Best Pract. Res. Clin. Rheumatol..

[3] Bergman S., Herrstrom P., Hogstrom K., Petersson I.F., Svensson B., Jacobsson L.T. (2001). Chronic musculoskeletal pain, prevalence rates, and sociodemographic associations in a Swedish population study. J. Rheumatol..

[4] Perez de Heredia-Torres M., Huertas-Hoyas E., Maximo-Bocanegra N., Palacios-Cena D., Fernandez-De-Las-Penas C. (2016). Cognitive performance in women with fibromyalgia: A case-control study. Aust. Occup. Ther. J..

[5] Aparicio V.A., Ortega F.B., Carbonell-Baeza A., Gatto-Cardia C., Sjostrom M., Ruiz J.R., Delgado-Fernandez M. (2013). Fibromyalgia’s key symptoms in normal-weight, overweight, and obese female patients. Pain Manag. Nurs..

[6] Wolfe F., Smythe H.A., Yunus M.B., Bennett R.M., Bombardier C., Goldenberg D.L., Tugwell P., Campbell S.M., Abeles M., Clark P. (1990). The American College of Rheumatology 1990 Criteria for the Classification of Fibromyalgia. Report of the Multicenter Criteria Committee. Arthritis Rheum..

[7] Breivik H., Collett B., Ventafridda V., Cohen R., Gallacher D. (2006). Survey of chronic pain in Europe: Prevalence, impact on daily life, and treatment. Eur. J. Pain.

[8] Tracey I., Woolf C.J., Andrews N.A. (2019). Composite Pain Biomarker Signatures for Objective Assessment and Effective Treatment. Neuron.

[9] Galvez-Sanchez C.M., Reyes Del Paso G.A. (2020). Diagnostic Criteria for Fibromyalgia: Critical Review and Future Perspectives. J. Clin. Med..

[10] Wåhlén K., Olausson P., Carlsson A., Ghafouri N., Gerdle B., Ghafouri B. (2017). Systemic alterations in plasma proteins from women with chronic widespread pain compared to healthy controls: A proteomic study. J. Pain Res..

[11] Olausson P., Gerdle B., Ghafouri N., Larsson B., Ghafouri B. (2012). Identification of proteins from interstitium of trapezius muscle in women with chronic myalgia using microdialysis in combination with proteomics. PLoS ONE.

[12] Hadrevi J., Bjorklund M., Kosek E., Hallgren S., Antti H., Fahlstrom M., Hellstrom F. (2015). Systemic differences in serum metabolome: A cross sectional comparison of women with localised and widespread pain and controls. Sci. Rep..

[13] Culic O., Cordero M.D., Zanic-Grubisic T., Somborac-Bacura A., Pucar L.B., Detel D., Varljen J., Barisic K. (2016). Serum activities of adenosine deaminase, dipeptidyl peptidase IV and prolyl endopeptidase in patients with fibromyalgia: Diagnostic implications. Clin. Rheumatol..

[14] Olausson P., Ghafouri B., Backryd E., Gerdle B. (2017). Clear differences in cerebrospinal fluid proteome between women with chronic widespread pain and healthy women—A multivariate explorative cross-sectional study. J. Pain Res..

[15] Zanette S.A., Dussan-Sarria J.A., Souza A., Deitos A., Torres I.L.S., Caumo W. (2014). Higher serum S100B and BDNF levels are correlated with a lower pressure-pain threshold in fibromyalgia. Mol. Pain.

[16] Bazzichi L., Ciregia F., Giusti L., Baldini C., Giannaccini G., Giacomelli C., Sernissi F., Bombardieri S., Lucacchini A. (2009). Detection of potential markers of primary fibromyalgia syndrome in human saliva. Proteom. Clin. Appl..

[17] Gerdle B., Ghafouri B., Ghafouri N., Backryd E., Gordh T. (2017). Signs of ongoing inflammation in female patients with chronic widespread pain: A multivariate, explorative, cross-sectional study of blood samples. Medicine.

[18] Jablochkova A., Backryd E., Kosek E., Mannerkorpi K., Ernberg M., Gerdle B., Ghafouri B. (2019). Unaltered low nerve growth factor and high brain-derived neurotrophic factor levels in plasma from patients with fibromyalgia after a 15-week progressive resistance exercise. J. Rehabil. Med..

[19] Gomez-Varela D., Barry A.M., Schmidt M. (2019). Proteome-based systems biology in chronic pain. J. Proteom..

[20] Niederberger E., Geisslinger G. (2008). Proteomics in neuropathic pain research. J. Am. Soc. Anesthesiol..

[21] Emilsson V., Ilkov M., Lamb J.R., Finkel N., Gudmundsson E.F., Pitts R., Hoover H., Gudmundsdottir V., Horman S.R., Aspelund T. (2018). Co-regulatory networks of human serum proteins link genetics to disease. Science.

[22] Manzoni C., Kia D.A., Vandrovcova J., Hardy J., Wood N.W., Lewis P.A., Ferrari R. (2018). Genome, transcriptome and proteome: The rise of omics data and their integration in biomedical sciences. Brief. Bioinform..

[23] Olausson P., Gerdle B., Ghafouri N., Sjostrom D., Blixt E., Ghafouri B. (2015). Protein alterations in women with chronic widespread pain--An explorative proteomic study of the trapezius muscle. Sci. Rep..

[24] Han C.L., Sheng Y.C., Wang S.Y., Chen Y.H., Kang J.H. (2020). Serum proteome profiles revealed dysregulated proteins and mechanisms associated with fibromyalgia syndrome in women. Sci. Rep..

[25] Wåhlén K., Ernberg M., Kosek E., Mannerkorpi K., Gerdle B., Ghafouri B. (2020). Significant correlation between plasma proteome profile and pain intensity, sensitivity, and psychological distress in women with fibromyalgia. Sci. Rep..

[26] Ramirez-Tejero J.A., Martinez-Lara E., Rus A., Camacho M.V., Del Moral M.L., Siles E. (2018). Insight into the biological pathways underlying fibromyalgia by a proteomic approach. J. Proteom..

[27] Olausson P., Ghafouri B., Ghafouri N., Gerdle B. (2016). Specific proteins of the trapezius muscle correlate with pain intensity and sensitivity—An explorative multivariate proteomic study of the trapezius muscle in women with chronic widespread pain. J. Pain Res..

[28] Wåhlén K., Ghafouri B., Ghafouri N., Gerdle B. (2018). Plasma Protein Pattern Correlates With Pain Intensity and Psychological Distress in Women With Chronic Widespread Pain. Front. Psychol..

[29] Kosek E., Cohen M., Baron R., Gebhart G.F., Mico J.A., Rice A.S., Rief W., Sluka A.K. (2016). Do we need a third mechanistic descriptor for chronic pain states?. Pain.

[30] Gerdle B., Bäckryd E., Novo M., Roeck Hansen E., Rothman M., Stålnacke B.-M., Westergren H., Rivano Fischer M. (2020). Smärtanalys—Diagnos, Smärtmekanismer, Psykologisk Och Social Bedömning.

[31] Nijs J., George S., Clauw D., Fernández-de-las-Peñas C., Kosek E., Ickmans K., Fernández-Carnero J., Polli A., Kapreli E., Huysmans E. (2021). Central sensitisation in chronic pain conditions: Latest discoveries and their potential for precision medicine. Lancet Rheumatol..

[32] Kosek E., Clauw D., Nijs J., Baron R., Gilron I., Harris R.E., Mico J.A., Rice A.S., Sterling M. (2021). Chronic nociplastic pain affecting the musculoskeletal system: Clinical criteria and grading system. Pain.

[33] Potvin S., Marchand S. (2016). Pain facilitation and pain inhibition during conditioned pain modulation in fibromyalgia and in healthy controls. Pain.

[34] Gerdle B., Wåhlén K., Ghafouri B. (2020). Plasma protein patterns are strongly correlated with pressure pain thresholds in women with chronic widespread pain and in healthy controls-an exploratory case-control study. Medicine.

[35] Muller M., Butikofer L., Andersen O.K., Heini P., Arendt-Nielsen L., Juni P., Curatolo M. (2021). Cold pain hypersensitivity predicts trajectories of pain and disability after low back surgery: A prospective cohort study. Pain.

[36] Gerdle B., Ghafouri B. (2020). Proteomic studies of common chronic pain conditions—A systematic review and associated network analyses. Expert Rev. Proteom..

[37] Wallin M., Liedberg G., Börsbo B., Gerdle B. (2012). Thermal detection and pain thresholds but not pressure pain thresholds are correlated with psychological factors in women with chronic whiplash-associated pain. Clin. J. Pain.

[38] Grundstrom H., Larsson B., Arendt-Nielsen L., Gerdle B., Kjolhede P. (2020). Pain catastrophizing is associated with pain thresholds for heat, cold and pressure in women with chronic pelvic pain. Scand. J. Pain.

[39] Gerdle B., Larsson B., Forsberg F., Ghafouri N., Karlsson L., Stensson N., Ghafouri B. (2014). Chronic widespread pain: Increased glutamate and lactate concentrations in the trapezius muscle and plasma. Clin. J. Pain.

[40] Gerdle B., Soderberg K., Salvador Puigvert L., Rosendal L., Larsson B. (2010). Increased interstitial concentrations of pyruvate and lactate in the trapezius muscle of patients with fibromyalgia: A microdialysis study. J. Rehabil. Med..

[41] Ghafouri N., Ghafouri B., Larsson B., Stensson N., Fowler C.J., Gerdle B. (2013). Palmitoylethanolamide and stearoylethanolamide levels in the interstitium of the trapezius muscle of women with chronic widespread pain and chronic neck-shoulder pain correlate with pain intensity and sensitivity. Pain.

[42] Ferreira-Valente M.A., Pais-Ribeiro J.L., Jensen M.P. (2011). Validity of four pain intensity rating scales. Pain.

[43] Zigmond A.S., Snaith R.P. (1983). The hospital anxiety and depression scale. Acta Psychiatr. Scand..

[44] LoMartire R., Äng B., Gerdle B., Vixner L. (2020). Psychometric properties of SF-36, EQ-5D and HADS in patients with chronic pain. Pain.

[45] Sullivan M.J.L., Bishop S.R., Pivik J. (1995). The Pain Catastrophizing Scale: Development and validation. Psychol. Assess..

[46] Burckhardt C.S., Anderson K.L. (2003). The Quality of Life Scale (QOLS): Reliability, validity, and utilization. Health Qual. Life Outcomes.

[47] Raak R., Wallin M. (2006). Thermal Thresholds and Catastrophizing in Individuals with Chronic Pain after Whiplash Injury. Biol. Res. Nurs..

[48] Wallin M.K., Raak R.I. (2008). Quality of life in subgroups of individuals with whiplash associated disorders. Eur. J. Pain.

[49] Fruhstorfer H., Lindblom U., Schmidt W. (1976). Method for quantitative estimation of thermal thresholds in patients. J. Neurol. Neurosurg. Psychiatry.

[50] Gorg A., Drews O., Luck C., Weiland F., Weiss W. (2009). 2-DE with IPGs. Electrophoresis.

[51] Wheelock A.M., Wheelock C.E. (2013). Trials and tribulations of omics data analysis: Assessing quality of SIMCA-based multivariate models using examples from pulmonary medicine. Mol. Biosyst..

[52] Eriksson L., Byrne T., Johansson E., Trygg J., Vikström C. (2013). Multi-and Megavariate Data Analysis: Basic Principles and Applications.

[53] Szklarczyk D., Gable A.L., Lyon D., Junge A., Wyder S., Huerta-Cepas J., Simonovic M., Doncheva N.T., Morris J.H., Bork P. (2019). STRING v11: Protein-protein association networks with increased coverage, supporting functional discovery in genome-wide experimental datasets. Nucleic Acids Res..

[54] Blumenstiel K., Gerhardt A., Rolke R., Bieber C., Tesarz J., Friederich H.C., Eich W., Treede R.D. (2011). Quantitative sensory testing profiles in chronic back pain are distinct from those in fibromyalgia. Clin. J. Pain.

[55] Hurtig I.M., Raak R.I., Kendall S.A., Gerdle B., Wahren L.K. (2001). Quantitative sensory testing in fibromyalgia patients and in healthy subjects: Identification of subgroups. Clin. J. Pain.

[56] Smith B.W., Tooley E.M., Montague E.Q., Robinson A.E., Cosper C.J., Mullins P.G. (2008). Habituation and sensitization to heat and cold pain in women with fibromyalgia and healthy controls. Pain.

[57] Brietzke A.P., Antunes L.C., Carvalho F., Elkifury J., Gasparin A., Sanches P.R.S., da Silva Junior D.P., Dussan-Sarria J.A., Souza A., da Silva Torres I.L. (2019). Potency of descending pain modulatory system is linked with peripheral sensory dysfunction in fibromyalgia: An exploratory study. Medicine.

[58] Rehm S., Sachau J., Hellriegel J., Forstenpointner J., Borsting Jacobsen H., Harten P., Gierthmuhlen J., Baron R. (2021). Pain matters for central sensitization: Sensory and psychological parameters in patients with fibromyalgia syndrome. Pain Rep..

[59] Grundstrom H., Gerdle B., Alehagen S., Bertero C., Arendt-Nielsen L., Kjolhede P. (2019). Reduced pain thresholds and signs of sensitization in women with persistent pelvic pain and suspected endometriosis. Acta Obstet. Gynecol. Scand..

[60] Tu Y., Zhang B., Cao J., Wilson G., Zhang Z., Kong J. (2019). Identifying inter-individual differences in pain threshold using brain connectome: A test-retest reproducible study. Neuroimage.

[61] Jin P., Lan J., Wang K., Baker M.S., Huang C., Nice E.C. (2018). Pathology, proteomics and the pathway to personalised medicine. Expert Rev. Proteom..

[62] Khoonsari P.E., Musunri S., Herman S., Svensson C.I., Tanum L., Gordh T., Kultima K. (2019). Systematic analysis of the cerebrospinal fluid proteome of fibromyalgia patients. J. Proteom..

[63] Bäckryd E., Tanum L., Lind A.L., Larsson A., Gordh T. (2017). Evidence of both systemic inflammation and neuroinflammation in fibromyalgia patients, as assessed by a multiplex protein panel applied to the cerebrospinal fluid and to plasma. J. Pain Res..

[64] Obreja O., Rukwied R., Nagler L., Schmidt M., Schmelz M., Namer B. (2018). Nerve growth factor locally sensitizes nociceptors in human skin. Pain.

[65] Yurkovich J.T., Hood L. (2019). Blood Is a Window into Health and Disease. Clin. Chem..

[66] Bruderer R., Muntel J., Muller S., Bernhardt O.M., Gandhi T., Cominetti O., Macron C., Carayol J., Rinner O., Astrup A. (2019). Analysis of 1508 Plasma Samples by Capillary-Flow Data-Independent Acquisition Profiles Proteomics of Weight Loss and Maintenance. Mol. Cell. Proteom..

[67] Baral P., Udit S., Chiu I.M. (2019). Pain and immunity: Implications for host defence. Nat. Rev. Immunol..

[68] Nguyen A.V., Soulika A.M. (2019). The Dynamics of the Skin’s Immune System. Int. J. Mol. Sci..

[69] Kabashima K., Honda T., Ginhoux F., Egawa G. (2019). The immunological anatomy of the skin. Nat. Rev. Immunol..

[70] Krausgruber T., Fortelny N., Fife-Gernedl V., Senekowitsch M., Schuster L.C., Lercher A., Nemc A., Schmidl C., Rendeiro A.F., Bergthaler A. (2020). Structural cells are key regulators of organ-specific immune responses. Nature.

[71] Rice F.L., Kruger L., Albrecht P.J., Gebhart G.F., Schmidt R.F. (2013). Histology of Nociceptors. Encyclopedia of Pain.

[72] Lacagnina M., Heijnen C., Watkins L., Grace P. (2021). Autoimmune regulation of chronic pain. Pain Rep..

[73] Abdo H., Calvo-Enrique L., Lopez J.M., Song J., Zhang M.D., Usoskin D., El Manira A., Adameyko I., Hjerling-Leffler J., Ernfors P. (2019). Specialized cutaneous Schwann cells initiate pain sensation. Science.

[74] Bray E.R., Cheret J., Yosipovitch G., Paus R. (2020). Schwann cells as underestimated, major players in human skin physiology and pathology. Exp. Dermatol..

[75] Karshikoff B., Tadros M., Mackey S., Zouikr I. (2019). Neuroimmune modulation of pain across the developmental spectrum. Curr. Opin. Behav. Sci..

[76] Chen L., Deng H., Cui H., Fang J., Zuo Z., Deng J., Li Y., Wang X., Zhao L. (2018). Inflammatory responses and inflammation-associated diseases in organs. Oncotarget.

[77] Coskun Benlidayi I. (2019). Role of inflammation in the pathogenesis and treatment of fibromyalgia. Rheumatol. Int..

[78] Goncalves Dos Santos G., Delay L., Yaksh T.L., Corr M. (2019). Neuraxial Cytokines in Pain States. Front. Immunol..

[79] Gerdle B., Forsgren M., Bengtsson A., Dahlqvist Leinhard O., Sören B., Karlsson A., Brandejsky V., Lund E., Lundberg P. (2013). Decreased muscle concentrations of ATP and PCR in the quadriceps muscle of fibromyalgia patients—A 31P MRS study. Eur. J. Pain.

[80] Park J.H., Phothimat P., Oates C.T., Hernanz-Schulman M., Olsen N.J. (1998). Use of P-31 magnetic resonance spectroscopy to detect metabolic abnormalities in muscles of patients with fibromyalgia. Arthritis Rheum..

[81] Gerdle B., Ghafouri B., Lund E., Bengtsson A., Lundberg P., Ettinger-Veenstra H.V., Leinhard O.D., Forsgren M.F. (2020). Evidence of Mitochondrial Dysfunction in Fibromyalgia: Deviating Muscle Energy Metabolism Detected Using Microdialysis and Magnetic Resonance. J. Clin. Med..

[82] Sluka K.A., Clauw D.J. (2016). Neurobiology of fibromyalgia and chronic widespread pain. Neuroscience.

[83] Schrepf A., Harper D.E., Harte S.E., Wang H., Ichesco E., Hampson J.P., Zubieta J.K., Clauw D.J., Harris R.E. (2016). Endogenous opioidergic dysregulation of pain in fibromyalgia: A PET and fMRI study. Pain.

[84] Üçeyler N., Sommer C., Häuser W., Perrot S. (2018). Small Nerve Fiber Pathology. Fibromylagia Syndrome and Widespread Pain—From Construction to Relevant Recognition.

[85] Gerdle B., Larsson B., Häuser W., Perrot S. (2018). Muscle. Fibromyalgia Syndrome and Widespread Pain—From Construction to Relevant Recognition.

[86] Albrecht D.S., Forsberg A., Sandstrom A., Bergan C., Kadetoff D., Protsenko E., Lampa J., Lee Y.C., Hoglund C.O., Catana C. (2019). Brain glial activation in fibromyalgia—A multi-site positron emission tomography investigation. Brain Behav. Immun..

[87] Jensen K.B., Kosek E., Petzke F., Carville S., Fransson P., Marcus H., Williams S.C.R., Choy E., Giesecke T., Mainguy Y. (2009). Evidence of dysfunctional pain inhibition in Fibromyalgia reflected in rACC during provoked pain. Pain.

[88] Littlejohn G., Guymer E. (2018). Neurogenic inflammation in fibromyalgia. Semin. Immunopathol..

[89] Van Ettinger-Veenstra H., Lundberg P., Alfoldi P., Sodermark M., Graven-Nielsen T., Sjors A., Engstrom M., Gerdle B. (2019). Chronic widespread pain patients show disrupted cortical connectivity in default mode and salience networks, modulated by pain sensitivity. J. Pain Res..

[90] Goubert D., Danneels L., Graven-Nielsen T., Descheemaeker F., Meeus M. (2017). Differences in Pain Processing Between Patients with Chronic Low Back Pain, Recurrent Low Back Pain, and Fibromyalgia. Pain Physician.

[91] Evdokimov D., Frank J., Klitsch A., Unterecker S., Warrings B., Serra J., Papagianni A., Saffer N., Meyer Zu Altenschildesche C., Kampik D. (2019). Reduction of skin innervation is associated with a severe fibromyalgia phenotype. Ann. Neurol..

[92] Fasolino A., Di Stefano G., Leone C., Galosi E., Gioia C., Lucchino B., Terracciano A., Di Franco M., Cruccu G., Truini A. (2020). Small-fibre pathology has no impact on somatosensory system function in patients with fibromyalgia. Pain.

[93] Sawaddiruk P., Paiboonworachat S., Chattipakorn N., Chattipakorn S.C. (2017). Alterations of brain activity in fibromyalgia patients. J. Clin. Neurosci..

[94] Iordanova Schistad E., Kong X.Y., Furberg A.S., Backryd E., Grimnes G., Emaus N., Rosseland L.A., Gordh T., Stubhaug A., Engdahl B. (2020). A population-based study of inflammatory mechanisms and pain sensitivity. Pain.

