# Supplementary material for: Thermal Pain Thresholds Are Significantly Associated with Plasma Proteins of the Immune System in Chronic Widespread Pain—An Exploratory Pilot Study Using Multivariate and Network Analyses
Source: J Clin Med. 2021 Aug 18;10(16):3652. doi: 10.3390/jcm10163652 (PMC8396857; doi:10.3390/jcm10163652)
Supplement: Supplementary file 1 [file jcm-10-03652-s001.zip › supplementary material _ two files/Supplementary text #2 final.pdf]

Supplementary text #2

Individual proteins

Content

Alpha-1-antitrypsin 3
Alpha-2-antiplasmin 3
Alpha-2-macroglobulin 3
Apolipoproteins 3
Beta-2-glycoprotein 1 4
Chemokine (C-C motif) ligand 19 (CCL19) 4
Chemokine (C-C motif) ligand 20 (CCL20; MIP-3alpha) 5
CUB-domain containing protein 1 (CDCP1) 5
Cluster of Differentiation 244 (CD244) 5
Ceruloplasmin 6
Clusterin 6
Complement factors 6
Creatine kinase M-type 8
Colony stimulating factor 1 (CSF-1) 8
EN-RAGE (Protein S100-A12) 8
Fibroblast growth factor 5 (FGF-5) 9
Fibroblast growth factor 21 (FGF-21) 9
FMS-like tyrosine kinase 3 ligand (Flt3L) 9
Fibrinogen alpha chain 9
Haptoglobin 10
Heat chock protein beta-1 10
Hemoglobin subunit alpha 10
Interleukin-7 (IL-7) 10
Interleukin 10 receptor, beta subunit (IL-10RB) 11
Immunoglobulins 11
Keratin, type II cytoskeletal I 12
Neurotrophin 3 (NT-3) 12
Phosphoglycerate mutase 2 13
Plasminogen 13
Serotransferrin 13
Tumor necrosis factor receptor superfamily member 9 (TNFRSF9) 13
Transthyretin (TTR) 14

Supplementary text #2

Vascular endothelial growth factor A (VEGF-A) 14
Vitamin D-binding protein 14
References 15

Supplementary text #2

Alpha-1-antitrypsin

This protein was a regressor of CPT in both groups. Increased expression of this positive acute phase protein has been reported in inflammation, infection, and in relation to different cytokines (beta-interferon, IL-1, IL-6 and TNF-alpha) and oxidative stress (Bergin et al. 2010). A primary function is to protect the tissue from enzymes released during inflammation besides this anti-inflammatory function also immunomodulatory, anti-infection and tissue repair related functions (Kim et al. 2018); some of these functions mainly arising from post-translational modifications (Lechowicz et al. 2020). This protein was upregulated in pressure ulcers in spinal cord injury patients, and in symptomatic apical periodontitis (Baldan-Martin et al. 2020, Loureiro et al. 2021). In a preliminary study of fibromyalgia (FM) patients an overexpression in serum was reported (Ruggiero et al. 2014).

Alpha-2-antiplasmin

This positive acute phase protein was a significant protein in chronic widespread pain (CWP) for both pain thresholds. Other proteoforms contributed to the multivariate differentiation between controls (CON) and CWP (increased in CWP) (Wåhlén et al. 2017). It (different proteoforms) was also identified in the regression of pain intensity and PPT in CWP (Wåhlén et al. 2018, Gerdle et al. 2020). Three proteoforms contributed to differentiate between FM and controls (one proteform increased in FM and two decreased) (Wåhlén et al. 2020). It also was positive regressor of pressure pain thresholds (PPT) and a negative regressor of psychological distress in FM (different proteoforms) (Wåhlén et al. 2020).

Alpha-2-macroglobulin

This positive acute phase protein has anti-inflammatory effects and was significant in the present four regressions (Cater et al. 2019). Increased blood levels have been reported in Alzheimer´s disease (AD) (Varma et al. 2017) and in type I diabetes (do Nascimento de Oliveira et al. 2018). The protein can inhibit proteases and influence signaling of and bind cytokines and growth factors including neurotrophins (e.g., IL-6, platelet-derived growth factor, nerve growth factor (NGF), tumor-necrosis factor (TNF)-α, and IL-1β) (Shimomura et al. 2018, Cater et al. 2019). The proteoforms differed between the two groups for cold pain thresholds (CPT) and for heat pain thresholds (HPT); for HPT, the signs also differed for the proteoforms. One of the proteoforms of alpha-2-macroglobulin was important in CON not only for CPT and HPT but also for PPT (Gerdle et al. 2020). Another proteoform correlated negatively with age in CON (Wåhlén et al. 2018).

Apolipoproteins

These proteins have functions in lipid metabolism but may also have a role in preventing the initiation of innate immunity i.e., anti-inflammatory role (Cho and Seong 2009, Gaglione et al. 2020). These proteins attenuate inflammation and enhance adaptive immunity and host defense (Borna et

al. 2019). We have earlier reported that apolipoprotein C-II was associated with PPT and psychological distress in CWP (Wåhlén et al. 2018, Gerdle et al. 2020) and a significantly increased plasma level was found in FM (Ramirez-Tejero et al. 2018).

Apolipoprotein A-I – i.e., the major structural protein of high-density lipoproteins (HDL) – is involved in regulation of cholesterol and also is involved in regulation of inflammatory and immune response (Georgila et al. 2019); generally it is considered as having anti-inflammatory properties but under certain conditions it may act pro-inflammatory. It was a significant protein in CWP for both pain thresholds. In a cohort of patients with FM we recently identified Apolipoprotein A-I as important for psychological distress (Wåhlén et al. 2020).

In CON apolipoprotein C-III was significant for CPT. Overexpression of this protein leads to hypertriglyceridemia and is considered as a risk factor for cardiovascular disease (CVD) (Yingchun et al. 2018, Taskinen et al. 2019). It also has a pro inflammatory role (Jin et al. 2016). This apolipoprotein can induce inflammation and organ damage by activating the NLRP3 inflammasome and hence functions as an alarmin triggering sterile inflammation (Zewinger et al. 2020). Increased plasma levels of apolipoprotein C-III has been found in diabetic peripheral neuropathy and was associated with BMI (Pek et al. 2017).

Beta-2-glycoprotein 1

This protein is the antigenic target of autoantibodies found in patients with antiphospholipid syndrome but it also is important in other aspects such as hemostasis, homeostasis and immunity (McDonnell et al. 2020). It is one of three proteins (besides C-reactive protein and thrombomodulin) with dual capability to up and down regulate the complement and coagulation systems depending upon external stimuli (McDonnell et al. 2020). The plasma protein correlated positively with HPT in CON. This protein (same proteoform) correlated positively with PPT and age in CON (Wåhlén et al. 2018, Gerdle et al. 2020). A negative correlation with pain intensity was found in peripheral neuropathic pain (Bäckryd et al. 2018).

Chemokine (C-C motif) ligand 19 (CCL19)

The chemokine CCL19 can have a dual role, promoting the immune response or having anti-inflammatory and immunosuppressive effects (Zou et al. 2016). It has been associated with several inflammatory conditions including atherosclerosis, rheumatoid arthritis, and multiple sclerosis (Kochumon et al. 2019). This chemokine correlated positively with CPT in CWP. The CCR 7 receptor and its ligands CCL19 and CCL21 control a diverse array of migratory events in adaptive immune function (Comerford et al. 2013). CCL19 gene expression in adipose tissue is increased in obese individuals and it was positively associated with proinflammatory substances e.g., IL-8, IL-12, IP-10, CCL5 etc. (Kochumon et al. 2019). Increased levels were found in serum of patients with lumbar

radicular pain one year after disc herniation (Moen et al. 2016). CCL19 was significantly increased in cerebrospinal fluid (CSF) in Osteoarthritis (OA) patients, in FM patients and in peripheral neuropathic pain patients (Bäckryd et al. 2017, Bäckryd et al. 2017, Palada et al. 2020). Neither in OA nor IN FM were found altered blood levels of CCL19 (Bäckryd et al. 2017, Palada et al. 2020). Moreover, this protein also correlated significantly with pain intensity in OA patients (Palada et al. 2020).

Chemokine (C-C motif) ligand 20 (CCL20; MIP-3alpha)

This chemokine was a significant regressor of HPT in CON. Depending on the condition the chemokine CCL20 may have both homeostatic and proinflammatory function (Jablonski et al. 2019). CCR6-CCL20 axis is an important modulator both of adaptive and immune responses in many inflammatory conditions (Ranasinghe and Eri 2019). Decreased serum levels was found in women with endometriosis (Jablonski et al. 2019). It contributed to the significant differentiation between CWP (increased level) and controls using plasma (Gerdle et al. 2017). Serum levels did not differ between knee OA patients and controls, but the levels correlated positively with disease severity within the patient group (Guan et al. 2019). Increased levels were found in serum of patients with lumbar radicular pain one year after disc herniation (Moen et al. 2016).

CUB-domain containing protein 1 (CDCP1)

In cancer increased levels of the cytokine CDCP1 are associated with progressive disease and markedly poorer survival (Predes et al. 2019, Khan et al. 2021). In OA patients and in patients with autoimmune endocrine diseases have been reported increased serum/plasma levels (Palada et al. 2020). CDCP1 can directly bind members of the TGF-beta superfamily and may hereby promote cancer progression and tumor cell metastasis (Predes et al. 2019). This cytokine correlated positively with HPT in CON. This protein was important for group differentiation between CWP (increased level) and CON (Gerdle et al. 2017). It was also positively correlated with pain intensity in CWP (Gerdle et al. 2017). The level in CSF correlated negatively with pain intensity in OA (Palada et al. 2020).

Cluster of Differentiation 244 (CD244)

This is a receptor also known as 2B4 receptor and SLAMF4 is member of the signaling lymphocyte activation molecule (SLAM), which is part of the Ig superfamily (Agresta et al. 2018, Buller et al. 2020). SLAM receptors are found on several immune cells that are involved in natural killer (NK) cell activation, which in turn are involved in e.g. recognition and clearance of cancer cells and infected cells (Buller et al. 2020). This protein correlated positively with CPT in CWP. It was also significantly increased in plasma in FM (Bäckryd et al. 2017). It was significantly increased in CSF in OA patients and was significantly associated with pain intensity in these patients (Palada et al. 2020).

Supplementary text #2

Ceruloplasmin

This positive acute phase protein is involved in the transport of copper. It was important in CON for both pain thresholds (several proteoforms, some similar). Elevated plasma levels of ceruloplasmin have been reported in the present CWP cohort compared to CON, in FM patients and in serum from lumbar disc herniation patients (La Rubia et al. 2013, Karabag et al. 2016, Wåhlén et al. 2017). One of the present proteoforms was significantly associated with pain intensity in CWP (Wåhlén et al. 2018). It (from plasma and serum) was a significant positive regressor of pain intensity in FM and in lumbar disc herniation pain patients (Karabag et al. 2016, Wåhlén et al. 2020).

Clusterin

Clusterin is a regulatory protein in energy homeostasis and inflammation (Ungsudechachai et al. 2020). Decreased levels of this protein in body fluids has been found in pain patients (Rodriguez-Rivera et al. 2021). It attenuates inflammation and it is suggested as a biomarker in various conditions (cancer, cardiovascular diseases, diabetes and metabolic syndrome) (Baralla et al. 2015). This protein has a neuroprotective role and it has been suggested to be protective versus oncologic pain (Rodriguez-Rivera et al. 2021). It also has been identified as potential candidate to treat neuropathic and inflammatory pain (Rodriguez-Rivera et al. 2021). Lower serum levels have been found in hand OA (Kropackova et al. 2018) and in degenerative scoliosis (Zhu et al. 2011). In healthy subjects clusterin plasma levels correlate with adiposity and systemic inflammation parameters and male sex (Won et al. 2014). The same proteoform was important in CWP for both thresholds. Another proteoform was important for HPT in CON but the sign differed from the situation for this protein in CWP. This protein but another proteoform correlated negatively with PPT in CWP (Gerdle et al. 2020) and positively with psychological distress (four proteoforms) in CWP (Wåhlén et al. 2018).

Complement factors

Complement factors were important in all four regressions. Complement and coagulation systems are important during the inflammatory process. Involvement of these systems has been reported in a serum proteomic studies of FM (Ramirez-Tejero et al. 2018, Han et al. 2020). The complement system is part of the humoral response within the innate immune system (Merle et al. 2015). This system identifies pathogens that are not recognized by antibodies (Totsch and Sorge 2017) and aid antibodies in clearing out damaged cells and microbes via the release of cytokines which in turn activate other cascades (Totsch and Sorge 2017). There are three activation pathways: 1) classical activation pathway, 2) lectin pathway and 3) the alternative pathway. Both our studies and studies of other researchers have reported involvement of the complement system in FM and in CWP (Wåhlén et al. 2017, Ramirez-Tejero et al. 2018, Han et al. 2020, Wåhlén et al. 2020). Moreover there is also reports that complement cascades are essential in neuropathic pain (Gu et al. 2019).

Supplementary text #2

Complement factor B
Complement factor B is an acute-phase protein and it is important for the complement activation via the alternative pathway (Ghafouri et al. 2016, Druart and Le Magueresse 2019). It correlated positively with CPT in CWP. The same proteoform was positively associated with psychological distress in the present CWP cohort (Wåhlén et al. 2018). In major depression upregulated complement factor B levels have been found (Wang et al. 2019). Another proteform of this protein correlated negatively with PPT. Still another another proteoform was important for group separation CWP vs. CON (Wåhlén et al. 2017). Complement factor B differed significantly between farmers with musculoskeletal disorders vs. controls (Ghafouri et al. 2016); increased levels were found in farmers with pain. The CSF level of this protein correlated negatively with pain intensity in neuropathic pain (Bäckryd et al. 2018).

Complement C3 alpha chain and Complement C3 alpha chain fragment 2
Complement C3 is an essential molecule for all three pathways of the complement system (Merle et al. 2015, Silawal et al. 2018, Druart and Le Magueresse 2019). Complement C3 consist of two chains (alpha and beta). This protein (different proteoforms) correlated positively with HPT in CON and positively with CPT in CWP. The same proteoform as in the regression of HPT in CON also correlated positively with PPT in CON. Another proteoform correlated positively with age in CON (Wåhlén et al. 2018). Complement C3 alpha chain fragment 2 correlated negatively with CPT in CWP. Complement C3 beta chain contributed to differentiation between FM and controls (Wåhlén et al. 2020).

Complement C4-B
This protein are affected both by the classical pathway and the lectin pathway (Druart and Le Magueresse 2019). Two proteoforms of this protein correlated positively with CPT in CON. One of these proteforms also correlated positively with BMI in CON (Wåhlén et al. 2018). Fragment of this protein contributed to the differentiation between FM and controls (Wåhlén et al. 2020).

Complement C1r subcomponent
This protein is part of the classical pathway of the complement system (Druart and Le Magueresse 2019). It correlated negatively with HPT both in CON and CWP but the proteoforms differed. These two proteoforms together with other proteoforms were increased in CWP and contributed to differentiation between CWP and CON (Wåhlén et al. 2017). In CWP two other proteoforms than the present correlated positively with pain intensity (Wåhlén et al. 2018). Other proteforms correlated positively with BMI in CON (Wåhlén et al. 2018). Two proteoforms were of importance for the differentiation between FM and controls; the levels were lower in the patients (Wåhlén et al. 2020). Another proteoform than the latter correlated negatively with psychological distress (Wåhlén et al. 2020).

Supplementary text #2

Complement component C7

This protein is together with C5b, C6, C8 and Cp involved in the membrane attack complex resulting in lysis of pathogens (Druart and Le Magueresse 2019). Two proteoforms correlated positively with HPT in CWP. One of the proteoforms correlated negatively with HADS (Wåhlén et al. 2018). This protein was also significantly upregulated in FM patients in a proteomic study of serum (Ramirez-Tejero et al. 2018). Down regulated CSF levels have been reported in neuropathic pain (Lind et al. 2019).

Creatine kinase M-type

Creatine kinases catalyze the conversion of creatine to phosphocreatine. Low muscle levels of ATP and phosphocreatine have been reported in FM (Park et al. 1998, Gerdle et al. 2013, Gerdle et al. 2020). This muscle protein correlated positively with CPT in CWP and negatively with HPT in CWP. It was upregulated in the interstitial fluid of the trapezius muscle in patients with trapezius myalgia (Olausson et al. 2012). A negative correlation existed between this protein (another proteoform) from a trapezius biopsy in CWP and pain intensity (Olausson et al. 2016). In a trauma experiment this protein was one of the most abundant proteins in the interstitial muscle fluid (Turkina et al. 2017).

Colony stimulating factor 1 (CSF-1)

This protein is also known as M-CSF is important for homeostasis and it is involved as a proinflammatory cytokine in different conditions that may be associated with pain such as arthritis, diabetes, multiple sclerosis and neurodegeneration (Saleh et al. 2018). CSF-1 correlated positively with HPT in CWP. The protein was up-regulated in saliva of neuropathic patients (Jonsson et al. 2021) and serum levels were dysregulated in knee OA patients and correlated positively with pain intensity in these patients (Giordano et al. 2020). Increased serum levels were found in lumbar radicular pain patients one year after disc herniation (Moen et al. 2016).

EN-RAGE (Protein S100-A12)

This inflammatory protein is highly abundant in neutrophils during acute inflammation and participates in immune regulation (Carvalho et al. 2020). The protein is involved in a broad array of digestive diseases e.g., gastroenteritis, gastric cancer, Crohn's disease, irritable bowel syndrome etc. (Carvalho et al. 2020). This was a significant protein in the regression of HPT in CON. It was also a significant plasma protein for the significant differentiation between FM and controls (Bäckryd et al. 2017); it was decreased in FM. Reduced levels were also noted in serum from patients with knee osteoarthritis (Giordano et al. 2020). Increased expression have been reported in endometriosis tissue (Sharma et al. 2010).

Supplementary text #2

Fibroblast growth factor 5 (FGF-5)

The Fibroblast growth factor family includes 22 structurally related polypeptides and they bind to four different receptors, which has been implicated in cell proliferation and differentiation during development and tissue repair (Chen et al. 2020). Fibroblast growth factor 5 (FGF-5) is a protein involved in in the regulation of the hair cycle in adults. Recent animal experiments indicate that it is an autocrine regulator of Schwann cell migration and adhesion (Chen et al. 2020). This protein correlated positively with HPT in CON. It was significantly increased in CSF in trigeminal neuralgia (Ericson et al. 2019). It also was significantly correlated with pain intensity in OA patients (Palada et al. 2020).

Fibroblast growth factor 21 (FGF-21)

FGF-21 - also a member of the Fibroblast growth factor family - is a hormone-like cytokine which regulates lipid metabolism and starvation response (Suomalainen et al. 2011, Tezze et al. 2019) (Chen et al. 2020). FGF-21 has been suggested as a biomarker for muscle-manifesting mitochondrial respiratory chain deficiencies (Suomalainen et al. 2011). This protein correlated negatively with HPT in CWP. In serum from OA patients it was increased and correlated with pain intensity (Giordano et al. 2020). It was also increased in serum and in CSF of OA patients (Palada et al. 2020).

FMS-like tyrosine kinase 3 ligand (Flt3L)

. It correlated negatively with HPT in CWP. This cytokine is involved in dendritic cell development and hence critical for the immune response (Durai et al. 2018). It increases the number of immune cells (lymphocytes (B cells and T cells)) by activating the hematopoietic progenitors. In women with interstitial cystitis no differences in urine level existed versus healthy controls (Abernethy et al. 2017). It was significantly decreased in peritoneal fluid in patients with endometriosis (Perricos et al. 2020). It was significantly increased in CSF and significantly decreased in serum of OA patients (Palada et al. 2020). Significantly higher CSF levels was noted in trigeminal neuralgia patients (Ericson et al. 2019).

Fibrinogen alpha chain

Fibrinogen – a positive acute phase protein - is a key regulator of inflammation and is elevated in response to inflammatory conditions (Borna et al. 2019). It is a dimer, where each monomer is composed of three non-identical chains, alpha, beta and gamma, linked together by several disulphide bonds (Mosesson et al. 2001). Fibrinogen alpha chain was a significant regressor of HPT in CWP. This substance is upregulated in women with endometriosis both in serum and in endometrial tissue (Chen et al. 2019). Upregulated levels have been found in various malignant neoplasms (Chen et al. 2019). Down regulated levels in serum have been reported in FM (Han et al. 2020) while another study reported increased levels in plasma (Wåhlén et al. 2018). It correlated negatively with

pain intensity in the present CWP and in FM (Wåhlén et al. 2018). In FM was also found a positive correlation with psychological distress (Wåhlén et al. 2018).

Haptoglobin

Haptoglobin was a negative regressor of CPT in CWP. Increased levels of the positive acute phase protein haptoglobin (Ramirez-Tejero et al. 2018, Borna et al. 2019) in serum and plasma have been reported in proteomic studies of FM/CWP (Wåhlén et al. 2017, Ramirez-Tejero et al. 2018, Wåhlén et al. 2020). This increase aims to counteract excessive oxidative stress. It was negatively associated with pain intensity and PPT in CWP, and negatively with psychological distress in FM (Wåhlén et al. 2018, Gerdle et al. 2020, Wåhlén et al. 2020).

Heat chock protein beta-1

This protective protein belongs to the small heat shock protein family. Heat shock proteins are chaperones and are induced in response to tissue and cellular stress and are important for facilitating protein synthesis, folding and assembly and environmental adaptation (Barbe et al. 2018). Exposure to inflammatory cytokines, oxidative stress etc. can induce this family of proteins (Ghayour-Mobarhan et al. 2009). Moreover, expression of this protein in response to stress is essential for survival of neurons (Katz et al. 2020). Increased levels of heat shock proteins have been associated with rheumatoid arthritis (Millar and Murrell 2012). This muscle protein correlated positively with CPT in CON. It contributed to differentiate between CWP and CON in our trapezius study (Olausson et al. 2015); the concentration was lower in patients.

Hemoglobin subunit alpha

This protein correlated negatively with CPT in CWP. Hemoglobin consists of four subunits, two alpha and two Beta. This protein was one of the most abundant proteins in the interstitial muscle fluid during the equilibration period and during post trauma in a trauma experiment of healthy women (Turkina et al. 2017).

Interleukin-7 (IL-7)

It correlated positively with CPT in CWP. Under disease conditions, the pro-inflammatory cytokine IL-7 mediates inflammation through several mechanisms and cell types (Willis et al. 2012, Taipa et al. 2019). Increased blood levels of IL-7 have been found in CRPS patients, in FM patients, and in a group of severe chronic pain patients (Alexander et al. 2012, Sturgill et al. 2014, Backryd et al. 2017, Hysing et al. 2019). It also has been associated with e.g. multiple sclerosis, RA and inflammatory bowel disease (Akdis et al. 2016). Two treatment studies have reported decreased level of IL-7 12 months post multimodal rehabilitation (Gerdle et al. 2019, Hysing et al. 2019).

Supplementary text #2

Interleukin 10 receptor, beta subunit (IL-10RB)

This protein correlated negatively with HPT in CWP; it is a subunit of the interleukin-10 receptor. IL-10 family receptors signal through Janus tyrosine kinases (JAKs) and signal transducers and activators of transcription (STATs) and are key anti-inflammatory cytokines (Baral et al. 2019, Wang et al. 2019). Under certain situations it can have pro-nociceptive properties (Goncalves Dos Santos et al. 2019). IL-10RB is ubiquitously expressed throughout the body (Wang et al. 2019). IL-10 correlated positively with pain intensity in CWP (Gerdle et al. 2017). Increased serum levels were found in lumbar radicular pain patients one year after disc herniation (Moen et al. 2016) but not in serum of OA patients (Palada et al. 2020). IL-10RB was significantly increased in CSF from OA patients and from trigeminal neuralgia patients (Ericson et al. 2019, Palada et al. 2020).

In a rehabilitation study changes in psychological distress were significantly and positively associated with pre-treatment values of IL-10 (Gerdle et al. 2019).

Immunoglobulins

The skin has an intricate network of immune cells which are important for host defense and tissue homeostasis (Nguyen and Soulika 2019). Animal studies report that structural cells of the skin (epithelial cells, endothelial cells and fibroblasts) express immune regulators and cytokine signaling and contribute to immunity (Krausgruber et al. 2020). Several studies including the present CWP cohort have reported the involvement of immunoglobulin parts in CWP/FM compared to controls even though not the same proteins as in the present study (Wåhlén et al. 2017, Ramirez-Tejero et al. 2018, Han et al. 2020, Wåhlén et al. 2020). B cells are stimulated by immunogens to differentiate into plasma cells; immunoglobulins (Ig) are produced by plasma cells. The immunogen or antigen reacts with a B-cell receptor (BCR) on the surface of B lymphocytes and a signal is produced that leads to activation of transcription factors to stimulate the synthesis of antibodies.

Ig light chain

Immunoglobulins (Igs) have two light chains and two heavy chains. Ig light chain (two proteoforms) correlated positively with HPT in CWP.

Ig kappa chain C region (IGKC)

This protein is the constant region of immunoglobulin light chains. IGKC correlated negatively with CPT in CWP. IGKC correlated negatively with HPT in CON. This protein contributed to the differentiation between CWP and CON (higher in CON) (Wåhlén et al. 2017). Another proteoform correlated negatively with age in CON (Wåhlén et al. 2018). IGKC further (two proteforms) contributed to the differentiation between FM and controls (higher in CON) (Wåhlén et al. 2020). In addition; IGKC (different proteforms) correlated positively with PPT and psychological distress in FM (Wåhlén et al. 2020). This protein was one of the most abundant proteins in the interstitial muscle

fluid in a trauma experiment in healthy women (Turkina et al. 2017). The serum expression of IGKC was found decreased in patients with obsessive-compulsive disorder (Zamanian Azodi et al. 2017).

Ig J chain

Ig J chain correlated negatively with CPT in CWP. This protein regulates polymer formation of IgA and IgM and modulate the secretory activity of these proteins (Ellias et al. 2012, Byron et al. 2020). Another proteform correlated negatively with age in CON (Wåhlén et al. 2018). This protein correlated positively with pain intensity in FM (Wåhlén et al. 2020). In saliva of patients with temporomandibular disorders myalgia lower levels was found (Jasim et al. 2020).

Ig alpha-2 chain C region

This protein is the constant region of Ig heavy chains. IG alpha-2 chain C region correlated positively with HPT in CON. It (same proteoform) also correlated positively with PPT in CON (Gerdle et al. 2020). Another proteoform contributed to differentiation between CWP and CON (decreased in patients) (Wåhlén et al. 2017). A proteoform correlated negatively with age in CON (Wåhlén et al. 2018). Another proteoform was weakly but significantly and positively associated with psychological distress in CWP (Wåhlén et al. 2018). This protein correlated positively with PPT in FM (Wåhlén et al. 2020). Upregulated plasma levels have been reported in colorectal cancer (Choi et al. 2013) and in type I and II diabetes (do Nascimento de Oliveira et al. 2018). This protein was significantly downregulated in plasma from patients with chronic rheumatic mitral stenosis (Mukherjee et al. 2014) and from patients with esophageal squamous cell carcinoma (Zhao et al. 2015).

Keratin, type II cytoskeletal I

This protein correlated positively with CPT in CON. This is a structural muscle protein belonging to the intermediate filament (IF) family and is important for the mitochondrial function (Shah et al. 2021). It contributed significantly to differentiate between CWP and controls in a proteomic study of trapezius (Olausson et al. 2015). This protein was one of the most abundant proteins in the interstitial muscle fluid during the equilibration period and post trauma in a trauma experiment (Turkina et al. 2017).

Neurotrophin 3 (NT-3)

This protein correlated negatively with HPT in CWP. Neurotrophins (NTs) regulate growth and apoptosis of neurons in the developing nervous system and repairing injured neurons. There are four NTs in humans: nerve growth factor (NGF), brain-derived neurotrophic factor (BDNF), neurotrophin 3 (NT-3), and neurotrophin 4 (NT-4). NTs bind with high affinity to their specific tyrosine receptor kinase (Trk); NGF binds to TrkA, BDNF and NT4 to TrkB, and NT3 to TrkC. All NTs can also bind with lower affinity to the neurotrophin receptor p75 (Khan and Smith 2015). Animal studies indicate that

Supplementary text #2

NT-3 can have both pro- and antinociceptive effects (Malfait et al. 2020). NGF and NT-3 are involved in sympathetic regulation (Fan et al. 2018).

Phosphoglycerate mutase 2

This protein correlated positively with HPT in CWP. A negative correlation existed between this protein (another proteoform) from a trapezius muscle biopsy in CWP and pain intensity (Olausson et al. 2016). Phosphoglycerate mutase (PGAM) catalyzes the reversible reaction of 3-phosphoglycerate (3-PGA) to 2-phosphoglycerate (2-PGA) in the glycolytic pathway (Tarnopolsky 2018). It is enriched in skeletal muscle, heart muscle and tongue.

Plasminogen

Plasminogen was significantly associated with CPT in both groups. Plasmin is released as a zymogen labelled plasminogen. Plasmin maintains blood flow by cleaving fibrin and breaking down intravascular clots or thrombi and is also important for tissue repair (Gibson et al. 2020). This positive acute phase protein is inactivated by proteins such as alpha-2-antiplasmin and alpha-2 macroglobulin. It (other proteoforms) was increased in CWP and among the important proteins for differentiating CWP from CON (Wåhlén et al. 2017). Four proteoforms of plasminogen also contributed to differentiate FM patients from healthy controls (Wåhlén et al. 2020). Plasminogen (different proteoforms) was also identified in the regressions of pain intensity and psychological distress in CWP (Wåhlén et al. 2018).

Serotransferrin

Serotransferrin was a significant regressor in CON for both thresholds. This negative acute phase protein is involved in binding and transportation of iron in the blood. It has been suggested that iron is involved in the pathophysiology of FM since it acts as a cofactor in the production of dopamine and serotonin (Ortancil et al. 2010). It (another proteoform than found in CON in the present study) contributed to multivariately differentiate between CWP and CON (Wåhlén et al. 2017), it was decreased in CWP. It also was positively correlated with PPT and psychological distress in CWP (Wåhlén et al. 2018, Gerdle et al. 2020). Two proteoforms of this protein contributed to differentiate between FM cohort and controls (Wåhlén et al. 2020); it was increased in FM. It was also a significant positive regressor of pain intensity in FM (Wåhlén et al. 2020). Significantly increased levels were found in farmers with pain conditions compared to controls (Ghafouri et al. 2016) and in a FM cohort (Ramirez-Tejero et al. 2018).

Tumor necrosis factor receptor superfamily member 9 (TNFRSF9)

This protein (also known as 4-1BB and CD137) correlated negatively with HPT in CWP. The ligand is TNFSF9 (4-1BBL) and animal studies have implicated 4-1BB – 4-1BBL interactions in the development of inflammatory disease (Croft et al. 2013). Moreover it has been suggested that 4-1BB has a pro-

inflammatory role (Croft et al. 2013). TNFRSF9 was significantly associated with incident distal sensorimotor polyneuropathy (Herder et al. 2018). It was significantly increased in CSF of OA patients and in these patients, it was significantly associated with pain intensity (Palada et al. 2020).

Transthyretin (TTR)

This protein correlated positively with CPT in CON. Transthyretin is a negative acute phase reactant in humans. Decreased serum concentrations have been found in liver disease, malnutrition and acute inflammation while elevated levels has been reported in lung neoplasms (Ding et al. 2014). We have reported that this protein contributed to differentiate between CWP and CON (decreased in patients)(Wåhlén et al. 2017). Moreover it correlated negatively with pain intensity in the present CWP cohort (Wåhlén et al. 2018). In a preliminary study of FM an overexpression in serum was reported (Ruggiero et al. 2014). It was increased in trigeminus neuralgia according to a proteomic study of plasma (Farajzadeh et al. 2018); the levels decreased after surgery.

Vascular endothelial growth factor A (VEGF-A)

It correlated negatively with HPT in CWP. This protein is regarded as a having both pro-nociceptive and neuroprotective effects (Hulse 2017, Llorian-Salvador and Gonzalez-Rodriguez 2018). VEGF-A increases healing, regeneration, revascularisation and it is increased in patients with lumbar radicular pain of severe intensity compared to patients with low pain intensity (Moen et al. 2016). A decrease in this substance in blood was observed in patients with disc herniation after epidural steroid injection (Weber et al. 2015). This protein decreased significantly in chronic pain patients after multidisciplinary pain rehabilitation (Gerdle et al. 2019). Plasma levels correlated negatively with pain intensity in neuropathic pain patients (Jonsson et al. 2021).

Vitamin D-binding protein

This protein (several proteoforms) was significant in both regressions in CWP and for HPT in CON. Vitamin D-binding protein - responsible for transport of vitamin D and its hydroxylated metabolites in plasma - also has metabolic roles e.g. transportation of fatty acids and endotoxin, and part of actin scavenging system and of the innate immunity (Giampaolino et al. 2019). Vitamin D-binding protein (spot no: 3408) was negatively associated with PPT in CON (Gerdle et al. 2020). This protein (another proteoform) was upregulated in CWP (Wåhlén et al. 2017) while farmers with pain had downregulated levels (Ghafouri et al. 2016). A systematic review concluded that patients with endometriosis have higher levels of this protein than controls (Sayegh et al. 2014); the literature was not in total consensus.

Supplementary text #2

References

Abernethy, M. G., A. Rosenfeld, J. R. White, M. G. Mueller, C. Lewicky-Gaupp and K. Kenton (2017). "Urinary Microbiome and Cytokine Levels in Women With Interstitial Cystitis." Obstet Gynecol **129**(3): 500-506.

Agresta, L., K. H. N. Hoebe and E. M. Janssen (2018). "The Emerging Role of CD244 Signaling in Immune Cells of the Tumor Microenvironment." Front Immunol **9**: 2809.

Akdis, M., A. Aab, C. Altunbulakli, K. Azkur, R. A. Costa, R. Crameri, S. Duan, T. Eiwegger, A. Eljaszewicz, R. Ferstl, R. Frei, M. Garbani, A. Globinska, L. Hess, C. Huitema, T. Kubo, Z. Komlosi, P. Konieczna, N. Kovacs, U. C. Kucuksezer, N. Meyer, H. Morita, J. Olzhausen, L. O'Mahony, M. Pezer, M. Prati, A. Rebane, C. Rhyner, A. Rinaldi, M. Sokolowska, B. Stanic, K. Sugita, A. Treis, W. van de Veen, K. Wanke, M. Wawrzyniak, P. Wawrzyniak, O. F. Wirz, J. S. Zakzuk and C. A. Akdis (2016). "Interleukins (from IL-1 to IL-38), interferons, transforming growth factor beta, and TNF-alpha: Receptors, functions, and roles in diseases." J Allergy Clin Immunol **138**(4): 984-1010.

Alexander, G. M., B. L. Peterlin, M. J. Perreault, J. R. Grothusen and R. J. Schwartzman (2012). "Changes in plasma cytokines and their soluble receptors in complex regional pain syndrome." J Pain **13**(1): 10-20.

Backryd, E., L. Tanum, A. L. Lind, A. Larsson and T. Gordh (2017). "Evidence of both systemic inflammation and neuroinflammation in fibromyalgia patients, as assessed by a multiplex protein panel applied to the cerebrospinal fluid and to plasma." J Pain Res **10**: 515-525.

Baldan-Martin, M., T. Martin-Rojas, N. Corbacho-Alonso, J. A. Lopez, T. Sastre-Oliva, F. Gil-Dones, J. Vazquez, J. M. Arevalo, L. Mourino-Alvarez and M. G. Barderas (2020). "Comprehensive Proteomic Profiling of Pressure Ulcers in Patients with Spinal Cord Injury Identifies a Specific Protein Pattern of Pathology." Adv Wound Care (New Rochelle) **9**(5): 277-294.

Baral, P., S. Udit and I. M. Chiu (2019). "Pain and immunity: implications for host defence." Nat Rev Immunol **19**(7): 433-447.

Baralla, A., E. Sotgiu, M. Deiana, S. Pasella, S. Pinna, A. Mannu, E. Canu, G. Sotgiu, A. Ganau, A. Zinellu, S. Sotgia, C. Carru and L. Deiana (2015). "Plasma Clusterin and Lipid Profile: A Link with Aging and Cardiovascular Diseases in a Population with a Consistent Number of Centenarians." PLoS One **10**(6): e0128029.

Barbe, M. F., D. M. Salvadeo and B. A. Hilliard (2018). Heat Shock Protein Response to Overuse Injuries. Regulation of Heat Shock Protein Responses. A. A. A. Asea and P. Kaur. Cham, Springer International Publishing: 277-292.

Bergin, D. A., E. P. Reeves, P. Meleady, M. Henry, O. J. McElvaney, T. P. Carroll, C. Condron, S. H. Chotirmall, M. Clynes, S. J. O'Neill and N. G. McElvaney (2010). "alpha-1 Antitrypsin regulates human neutrophil chemotaxis induced by soluble immune complexes and IL-8." J Clin Invest **120**(12): 4236-4250.

Borna, H., S. H. Hosseini Qale Noe, A. B. Harchegani, N. R. Talatappe, M. Ghatrehsamani, M. Ghanei and A. Shahriary (2019). "A review on proteomics analysis to reveal biological pathways and predictive proteins in sulfur mustard exposed patients: roles of inflammation and oxidative stress." Inhal Toxicol **31**(1): 3-11.

Buller, C. W., P. A. Mathew and S. O. Mathew (2020). "Roles of NK Cell Receptors 2B4 (CD244), CS1 (CD319), and LLT1 (CLEC2D) in Cancer." Cancers (Basel) **12**(7).

Byron, M. J., D. C. Koester, K. L. Edwards, P. E. Mozdziak, C. E. Farin and A. E. Crosier (2020). "Immunoglobulin J chain as a non-invasive indicator of pregnancy in the cheetah (Acinonyx jubatus)." PLoS One **15**(2): e0225354.

Bäckryd, E., S. Edstrom, B. Gerdle and B. Ghafouri (2018). "Do fragments and glycosylated isoforms of alpha-1-antitrypsin in CSF mirror spinal pathophysiological mechanisms in chronic peripheral neuropathic pain? An exploratory, discovery phase study." BMC Neurol **18**(1): 116.

Bäckryd, E., A. L. Lind, M. Thulin, A. Larsson, B. Gerdle and T. Gordh (2017). "High levels of cerebrospinal fluid chemokines point to the presence of neuroinflammation in peripheral

neuropathic pain: a cross-sectional study of 2 cohorts of patients compared with healthy controls." Pain **158**(12): 2487-2495.
Bäckryd, E., L. Tanum, A. L. Lind, A. Larsson and T. Gordh (2017). "Evidence of both systemic inflammation and neuroinflammation in fibromyalgia patients, as assessed by a multiplex protein panel applied to the cerebrospinal fluid and to plasma." J Pain Res **10**: 515-525.
Carvalho, A., J. Lu, J. D. Francis, R. E. Moore, K. P. Haley, R. S. Doster, S. D. Townsend, J. G. Johnson, S. M. Damo and J. A. Gaddy (2020). "S100A12 in Digestive Diseases and Health: A Scoping Review." Gastroenterol Res Pract **2020**: 2868373.
Cater, J. H., M. R. Wilson and A. R. Wyatt (2019). "Alpha-2-Macroglobulin, a Hypochlorite-Regulated Chaperone and Immune System Modulator." Oxid Med Cell Longev **2019**: 5410657.
Chen, B., R. Hu, Q. Min, Y. Li, D. B. Parkinson and X. P. Dun (2020). "FGF5 Regulates Schwann Cell Migration and Adhesion." Front Cell Neurosci **14**: 237.
Chen, Y., H. Li, H. Y. Cheng, M. Rui-Qiong, X. Ye, H. Cui, Z. Hong-Lan and X. H. Chang (2019). "Fibrinogen alpha chain is up-regulated and affects the pathogenesis of endometriosis." Reprod Biomed Online **39**(6): 893-904.
Cho, N. H. and S. Y. Seong (2009). "Apolipoproteins inhibit the innate immunity activated by necrotic cells or bacterial endotoxin." Immunology **128**(1 Suppl): e479-486.
Choi, J. W., H. Liu, D. H. Shin, G. I. Yu, J. S. Hwang, E. S. Kim and J. W. Yun (2013). "Proteomic and cytokine plasma biomarkers for predicting progression from colorectal adenoma to carcinoma in human patients." Proteomics **13**(15): 2361-2374.
Comerford, I., Y. Harata-Lee, M. D. Bunting, C. Gregor, E. E. Kara and S. R. McColl (2013). "A myriad of functions and complex regulation of the CCR7/CCL19/CCL21 chemokine axis in the adaptive immune system." Cytokine Growth Factor Rev **24**(3): 269-283.
Croft, M., C. A. Benedict and C. F. Ware (2013). "Clinical targeting of the TNF and TNFR superfamilies." Nat Rev Drug Discov **12**(2): 147-168.
Ding, H., J. Liu, R. Xue, P. Zhao, Y. Qin, F. Zheng and X. Sun (2014). "Transthyretin as a potential biomarker for the differential diagnosis between lung cancer and lung infection." Biomed Rep **2**(5): 765-769.
do Nascimento de Oliveira, V., A. B. M. Lima-Neto, M. F. van Tilburg, A. C. de Oliveira Monteiro-Moreira, M. Duarte Pinto Lobo, D. Rondina, V. O. Fernandes, A. Montenegro, R. M. J. Montenegro and M. I. F. Guedes (2018). "Proteomic analysis to identify candidate biomarkers associated with type 1 diabetes." Diabetes Metab Syndr Obes **11**: 289-301.
Druart, M. and C. Le Magueresse (2019). "Emerging Roles of Complement in Psychiatric Disorders." Front Psychiatry **10**: 573.
Durai, V., P. Bagadia, C. G. Briseno, D. J. Theisen, A. Iwata, J. T. t. Davidson, M. Gargaro, D. H. Fremont, T. L. Murphy and K. M. Murphy (2018). "Altered compensatory cytokine signaling underlies the discrepancy between Flt3(-/-) and Flt3l(-/-) mice." J Exp Med **215**(5): 1417-1435.
Ellias, M. F., S. H. Zainal Ariffin, S. A. Karsani, M. Abdul Rahman, S. Senafi and R. Megat Abdul Wahab (2012). "Proteomic analysis of saliva identifies potential biomarkers for orthodontic tooth movement." ScientificWorldJournal **2012**: 647240.
Ericson, H., S. Abu Hamdeh, E. Freyhult, F. Stiger, E. Backryd, A. Svenningsson, T. Gordh and K. Kultima (2019). "Cerebrospinal fluid biomarkers of inflammation in trigeminal neuralgia patients operated with microvascular decompression." Pain **160**(11): 2603-2611.
Fan, W., X. Zhu, Y. He, H. Li, W. Gu, F. Huang and H. He (2018). "Peripheral sympathetic mechanisms in orofacial pain." J Pain Res **11**: 2425-2431.
Farajzadeh, A., S. Z. Bathaie, J. Arabkheradmand, S. M. Ghodsi and S. Faghihzadeh (2018). "Different Pain States of Trigeminal Neuralgia Make Significant Changes in the Plasma Proteome and Some Biochemical Parameters: a Preliminary Cohort Study." J Mol Neurosci **66**(4): 524-534.
Gaglione, R., E. Pizzo, E. Notomista, C. de la Fuente-Nunez and A. Arciello (2020). "Host Defence Cryptides from Human Apolipoproteins: Applications in Medicinal Chemistry." Curr Top Med Chem **20**(14): 1324-1337.

Supplementary text #2

Georgila, K., D. Vyrla and E. Drakos (2019). "Apolipoprotein A-I (ApoA-I), Immunity, Inflammation and Cancer." Cancers (Basel) **11**(8).

Gerdle, B., E. Bäckryd, T. Falkenberg, E. Lundstrom and B. Ghafouri (2019). "Changes in inflammatory plasma proteins from patients with chronic pain associated with treatment in an interdisciplinary multimodal rehabilitation program - an explorative multivariate pilot study." Scand J Pain **20**(1): 125-138.

Gerdle, B., M. Forsgren, A. Bengtsson, O. Dahlqvist Leinhard, B. Sören, A. Karlsson, V. Brandejsky, E. Lund and P. Lundberg (2013). "Decreased muscle concentrations of ATP and PCR in the quadriceps muscle of fibromyalgia patients - a 31P MRS study." Eur J Pain **17**: 12-15-1215.

Gerdle, B., B. Ghafouri, N. Ghafouri, E. Backryd and T. Gordh (2017). "Signs of ongoing inflammation in female patients with chronic widespread pain: A multivariate, explorative, cross-sectional study of blood samples." Medicine (Baltimore) **96**(9): e6130.

Gerdle, B., B. Ghafouri, E. Lund, A. Bengtsson, P. Lundberg, H. V. Ettinger-Veenstra, O. D. Leinhard and M. F. Forsgren (2020). "Evidence of Mitochondrial Dysfunction in Fibromyalgia: Deviating Muscle Energy Metabolism Detected Using Microdialysis and Magnetic Resonance." J Clin Med **9**(11): 3527.

Gerdle, B., K. Wåhlén and B. Ghafouri (2020). "Plasma protein patterns are strongly correlated with pressure pain thresholds in women with chronic widespread pain and in healthy controls-an exploratory case-control study." Medicine (Baltimore) **99**(22): e20497.

Ghafouri, B., A. Carlsson, S. Holmberg, A. Thelin and C. Tagesson (2016). "Biomarkers of systemic inflammation in farmers with musculoskeletal disorders; a plasma proteomic study." BMC Musculoskelet Disord **17**(1): 206.

Ghayour-Mobarhan, M., A. A. Rahsepar, S. Tavallaie, S. Rahsepar and G. A. Ferns (2009). "The potential role of heat shock proteins in cardiovascular disease: evidence from in vitro and in vivo studies." Adv Clin Chem **48**: 27-72.

Giampaolino, P., L. Della Corte, V. Foreste and G. Bifulco (2019). "Is there a relationship between Vitamin D and Endometriosis? An overview of literature." Curr Pharm Des.

Gibson, B. H. Y., M. T. Duvernay, S. N. Moore-Lotridge, M. J. Flick and J. G. Schoenecker (2020). "Plasminogen activation in the musculoskeletal acute phase response: Injury, repair, and disease." Res Pract Thromb Haemost **4**(4): 469-480.

Giordano, R., K. K. Petersen, H. H. Andersen, O. Simonsen and L. Arendt-Nielsen (2020). "Serum Inflammatory Markers in Patients With Knee Osteoarthritis: A Proteomic Approach." Clin J Pain **36**(4): 229-237.

Goncalves Dos Santos, G., L. Delay, T. L. Yaksh and M. Corr (2019). "Neuraxial Cytokines in Pain States." Front Immunol **10**: 3061.

Gu, Y., Z. Qiu, N. Cheng, C. Chen, Z. Hei and X. Li (2019). "Identification of potential mechanism and hub genes for neuropathic pain by expression-based genome-wide association study." J Cell Biochem **120**(4): 4912-4923.

Guan, J., Y. Li, L. B. Ding, G. Y. Liu, X. F. Zheng, W. Xue and H. J. Wang (2019). "Relationship between serum and synovial fluid CCL20 concentrations with disease severity in primary knee osteoarthritis." J Musculoskelet Neuronal Interact **19**(3): 326-332.

Han, C. L., Y. C. Sheng, S. Y. Wang, Y. H. Chen and J. H. Kang (2020). "Serum proteome profiles revealed dysregulated proteins and mechanisms associated with fibromyalgia syndrome in women." Sci Rep **10**(1): 12347.

Herder, C., J. M. Kannenberg, M. Carstensen-Kirberg, A. Strom, G. J. Bonhof, W. Rathmann, C. Huth, W. Koenig, M. Heier, J. Krumsiek, A. Peters, C. Meisinger, M. Roden, B. Thorand and D. Ziegler (2018). "A Systemic Inflammatory Signature Reflecting Cross Talk Between Innate and Adaptive Immunity Is Associated With Incident Polyneuropathy: KORA F4/FF4 Study." Diabetes **67**(11): 2434-2442.

Hulse, R. P. (2017). "Role of VEGF-A in chronic pain." Oncotarget **8**(7): 10775-10776.

Supplementary text #2

Hysing, E. B., L. Smith, M. Thulin, R. Karlsten, K. Bothelius and T. Gordh (2019). "Detection of systemic inflammation in severely impaired chronic pain patients and effects of a multimodal pain rehabilitation program." Scand J Pain **19**(2): 235-244.

Jablonski, G., B. Sobkiewicz, M. Kaczmarek, A. Krygier, J. Bienkiewicz and A. Malinowski (2019). "Serum concentrations of CCL20/MIP-3alpha in women with endometriosis." Prz Menopauzalny **18**(1): 9-14.

Jasim, H., M. Ernberg, A. Carlsson, B. Gerdle and B. Ghafouri (2020). "Protein Signature in Saliva of Temporomandibular Disorders Myalgia." Int J Mol Sci **21**(7).

Jin, J. L., Y. L. Guo and J. J. Li (2016). "Apoprotein C-III: A review of its clinical implications." Clin Chim Acta **460**: 50-54.

Jonsson, M., B. Gerdle, B. Ghafouri and E. Backryd (2021). "The inflammatory profile of cerebrospinal fluid, plasma, and saliva from patients with severe neuropathic pain and healthy controls-a pilot study." BMC Neurosci **22**(1): 6.

Karabag, H., A. Yetisgin, E. Savik, M. Kilic, R. Aridici, A. Taskin and T. Ulas (2016). "Evaluation of ceruloplasmin levels in patients with lumbar disc herniation." J Back Musculoskelet Rehabil.

Katz, M., M. Davis, F. C. Garton, R. Henderson, V. Bharti, N. Wray and P. McCombe (2020). "Mutations in heat shock protein beta-1 (HSPB1) are associated with a range of clinical phenotypes related to different patterns of motor neuron dysfunction: A case series." J Neurol Sci **413**: 116809.

Khan, N. and M. T. Smith (2015). "Neurotrophins and Neuropathic Pain: Role in Pathobiology." Molecules **20**(6): 10657-10688.

Khan, T., T. Kryza, N. Lyons, Y. He and J. Hooper (2021). "The CDCP1 signalling hub: a target for cancer detection and therapeutic intervention." Cancer Res **Epub ahead of print**.

Kim, M., Q. Cai and Y. Oh (2018). "Therapeutic potential of alpha-1 antitrypsin in human disease." Ann Pediatr Endocrinol Metab **23**(3): 131-135.

Kochumon, S., F. Al-Rashed, M. Abu-Farha, S. Devarajan, J. Tuomilehto and R. Ahmad (2019). "Adipose tissue expression of CCL19 chemokine is positively associated with insulin resistance." Diabetes Metab Res Rev **35**(2): e3087.

Krausgruber, T., N. Fortelny, V. Fife-Gernedl, M. Senekowitsch, L. C. Schuster, A. Lercher, A. Nemc, C. Schmidl, A. F. Rendeiro, A. Bergthaler and C. Bock (2020). "Structural cells are key regulators of organ-specific immune responses." Nature **583**(7815): 296-302.

Kropackova, T., O. Sleglova, O. Ruzickova, J. Vencovsky, K. Pavelka and L. Senolt (2018). "Lower serum clusterin levels in patients with erosive hand osteoarthritis are associated with more pain." BMC Musculoskelet Disord **19**(1): 264.

La Rubia, M., A. Rus, F. Molina and M. L. Del Moral (2013). "Is fibromyalgia-related oxidative stress implicated in the decline of physical and mental health status?" Clin Exp Rheumatol **31**(6 Suppl 79): S121-127.

Lechowicz, U., S. Rudzinski, A. Jezela-Stanek, S. Janciauskiene and J. Chorostowska-Wynimko (2020). "Post-Translational Modifications of Circulating Alpha-1-Antitrypsin Protein." Int J Mol Sci **21**(23).

Lind, A. L., D. Just, M. Mikus, C. Fredolini, M. Ioannou, B. Gerdle, B. Ghafouri, E. Backryd, L. Tanum, T. Gordh and A. Manberg (2019). "CSF levels of apolipoprotein C1 and autotaxin found to associate with neuropathic pain and fibromyalgia." J Pain Res **12**: 2875-2889.

Llorian-Salvador, M. and S. Gonzalez-Rodriguez (2018). "Painful Understanding of VEGF." Front Pharmacol **9**: 1267.

Loureiro, C., M. A. R. Buzalaf, F. R. N. Moraes, T. M. O. Ventura, V. T. Pela, J. P. Pessan and R. C. Jacinto (2021). "Quantitative proteomic analysis in symptomatic and asymptomatic apical periodontitis." Int Endod J.

Malfait, A. M., R. E. Miller and J. A. Block (2020). "Targeting neurotrophic factors: Novel approaches to musculoskeletal pain." Pharmacol Ther **211**: 107553.

McDonnell, T., C. Wincup, I. Buchholz, C. Pericleous, I. Giles, V. Ripoll, H. Cohen, M. Delcea and A. Rahman (2020). "The role of beta-2-glycoprotein I in health and disease associating structure with function: More than just APS." Blood Rev **39**: 100610.

Supplementary text #2

Merle, N. S., R. Noe, L. Halbwachs-Mecarelli, V. Fremeaux-Bacchi and L. T. Roumenina (2015). "Complement System Part II: Role in Immunity." Front Immunol **6**: 257.

Millar, N. L. and G. A. Murrell (2012). "Heat shock proteins in tendinopathy: novel molecular regulators." Mediators Inflamm **2012**: 436203.

Moen, A., A. L. Lind, M. Thulin, M. Kamali-Moghaddam, C. Roe, J. Gjerstad and T. Gordh (2016). "Inflammatory Serum Protein Profiling of Patients with Lumbar Radicular Pain One Year after Disc Herniation." Int J Inflam **2016**: 3874964.

Mosesson, M. W., K. R. Siebenlist and D. A. Meh (2001). "The structure and biological features of fibrinogen and fibrin." Ann N Y Acad Sci **936**: 11-30.

Mukherjee, S., M. G. Jagadeeshaprasad, T. Banerjee, S. K. Ghosh, M. Biswas, S. Dutta, M. J. Kulkarni, S. Pattari and A. Bandyopadhyay (2014). "Proteomic analysis of human plasma in chronic rheumatic mitral stenosis reveals proteins involved in the complement and coagulation cascade." Clin Proteomics **11**(1): 35.

Nguyen, A. V. and A. M. Soulika (2019). "The Dynamics of the Skin's Immune System." Int J Mol Sci **20**(8).

Olausson, P., B. Gerdle, N. Ghafouri, B. Larsson and B. Ghafouri (2012). "Identification of proteins from interstitium of trapezius muscle in women with chronic myalgia using microdialysis in combination with proteomics." PLoS One **7**(12): e52560.

Olausson, P., B. Gerdle, N. Ghafouri, D. Sjostrom, E. Blixt and B. Ghafouri (2015). "Protein alterations in women with chronic widespread pain--An explorative proteomic study of the trapezius muscle." Sci Rep **5**: 11894.

Olausson, P., B. Ghafouri, N. Ghafouri and B. Gerdle (2016). "Specific proteins of the trapezius muscle correlate with pain intensity and sensitivity - an explorative multivariate proteomic study of the trapezius muscle in women with chronic widespread pain." J Pain Res **9**: 345-356.

Ortancil, O., A. Sanli, R. Eryuksel, A. Basaran and H. Ankarali (2010). "Association between serum ferritin level and fibromyalgia syndrome." Eur J Clin Nutr **64**(3): 308-312.

Palada, V., A. S. Ahmed, E. Freyhult, A. Hugo, K. Kultima, C. I. Svensson and E. Kosek (2020). "Elevated inflammatory proteins in cerebrospinal fluid from patients with painful knee osteoarthritis are associated with reduced symptom severity." J Neuroimmunol **349**: 577391.

Park, J. H., P. Phothimat, C. T. Oates, M. Hernanz-Schulman and N. J. Olsen (1998). "Use of P-31 magnetic resonance spectroscopy to detect metabolic abnormalities in muscles of patients with fibromyalgia." Arthritis Rheum **41**(3): 406-413.

Pek, S. L. T., C. F. Sum, L. Y. Yeoh, S. B. M. Lee, W. E. Tang, S. C. Lim and S. Tavintharan (2017). "Association of apolipoprotein-CIII (apoC-III), endothelium-dependent vasodilation and peripheral neuropathy in a multi-ethnic population with type 2 diabetes." Metabolism **72**: 75-82.

Perricos, A., R. Wenzl, H. Husslein, T. Eiwegger, M. Gstoettner, A. Weinhaeusel, G. Beikircher and L. Kuessel (2020). "Does the Use of the "Proseek((R)) Multiplex Oncology I Panel" on Peritoneal Fluid Allow a Better Insight in the Pathophysiology of Endometriosis, and in Particular Deep-Infiltrating Endometriosis?" J Clin Med **9**(6).

Predes, D., J. V. R. Cruz, J. G. Abreu and F. A. Mendes (2019). "CUB domain-containing protein 1 (CDCP1) binds transforming growth factor beta family members and increase TGF-beta1 signaling pathway." Exp Cell Res **383**(1): 111499.

Ramirez-Tejero, J. A., E. Martinez-Lara, A. Rus, M. V. Camacho, M. L. Del Moral and E. Siles (2018). "Insight into the biological pathways underlying fibromyalgia by a proteomic approach." J Proteomics **186**: 47-55.

Ranasinghe, R. and R. Eri (2019). "CCR6-CCL20-mediated immunological pathways in inflammatory bowel disease." Gastrointest Disord **1**: 15-29.

Rodriguez-Rivera, C., M. M. Garcia, M. Molina-Alvarez, C. Gonzalez-Martin and C. Goicoechea (2021). "Clusterin: Always protecting. Synthesis, function and potential issues." Biomed Pharmacother **134**: 111174.

Ruggiero, V., B. Era, E. Cacace, L. Molin, M. Corda, A. Fais and S. Utzeri (2014). "A preliminary study on serum proteomics in fibromyalgia syndrome." Clin Chem Lab Med **52**(9): e207-210.

Supplementary text #2

Saleh, R., M. C. Lee, S. H. Khiew, C. Louis, A. J. Fleetwood, A. Achuthan, I. Forster, A. D. Cook and J. A. Hamilton (2018). "CSF-1 in Inflammatory and Arthritic Pain Development." J Immunol **201**(7): 2042-2053.

Sayegh, L., H. Fuleihan Gel and A. H. Nassar (2014). "Vitamin D in endometriosis: a causative or confounding factor?" Metabolism **63**(1): 32-41.

Shah, M., L. A. Chacko, J. P. Joseph and V. Ananthanarayanan (2021). "Mitochondrial dynamics, positioning and function mediated by cytoskeletal interactions." Cell Mol Life Sci.

Sharma, I., V. Dhawan, S. C. Saha, B. Rashmi and L. K. Dhaliwal (2010). "Implication of the RAGE-EN-RAGE axis in endometriosis." Int J Gynaecol Obstet **110**(3): 199-202.

Shimomura, R., T. Nezu, N. Hosomi, S. Aoki, T. Sugimoto, N. Kinoshita, M. Araki, T. Takahashi, H. Maruyama and M. Matsumoto (2018). "Alpha-2-macroglobulin as a Promising Biological Marker of Endothelial Function." J Atheroscler Thromb **25**(4): 350-358.

Silawal, S., J. Triebel, T. Bertsch and G. Schulze-Tanzil (2018). "Osteoarthritis and the Complement Cascade." Clin Med Insights Arthritis Musculoskelet Disord **11**: 1179544117751430.

Sturgill, J., E. McGee and V. Menzies (2014). "Unique cytokine signature in the plasma of patients with fibromyalgia." J Immunol Res **2014**: 938576.

Suomalainen, A., J. M. Elo, K. H. Pietilainen, A. H. Hakonen, K. Sevastianova, M. Korpela, P. Isohanni, S. K. Marjavaara, T. Tyni, S. Kiuru-Enari, H. Pihko, N. Darin, K. Ounap, L. A. Kluijtmans, A. Paetau, J. Buzkova, L. A. Bindoff, J. Annunen-Rasila, J. Uusimaa, A. Rissanen, H. Yki-Jarvinen, M. Hirano, M. Tulinius, J. Smeitink and H. Tyynismaa (2011). "FGF-21 as a biomarker for muscle-manifesting mitochondrial respiratory chain deficiencies: a diagnostic study." Lancet Neurol **10**(9): 806-818.

Taipa, R., S. P. das Neves, A. L. Sousa, J. Fernandes, C. Pinto, A. P. Correia, E. Santos, P. S. Pinto, P. Carneiro, P. Costa, D. Santos, I. Alonso, J. Palha, F. Marques, S. Cavaco and N. Sousa (2019). "Proinflammatory and anti-inflammatory cytokines in the CSF of patients with Alzheimer's disease and their correlation with cognitive decline." Neurobiol Aging **76**: 125-132.

Tarnopolsky, M. A. (2018). "Myopathies Related to Glycogen Metabolism Disorders." Neurotherapeutics **15**(4): 915-927.

Taskinen, M. R., C. J. Packard and J. Boren (2019). "Emerging Evidence that ApoC-III Inhibitors Provide Novel Options to Reduce the Residual CVD." Curr Atheroscler Rep **21**(8): 27.

Tezze, C., V. Romanello and M. Sandri (2019). "FGF21 as Modulator of Metabolism in Health and Disease." Front Physiol **10**: 419.

Totsch, S. K. and R. E. Sorge (2017). "Immune System Involvement in Specific Pain Conditions." Mol Pain **13**: 1744806917724559.

Turkina, M. V., N. Ghafouri, B. Gerdle and B. Ghafouri (2017). "Evaluation of dynamic changes in interstitial fluid proteome following microdialysis probe insertion trauma in trapezius muscle of healthy women." Sci Rep **7**: 43512.

Ungsudechachai, T., S. Honsawek, J. Jittikoon and W. Udomsinprasert (2020). "Clusterin Is Associated with Systemic and Synovial Inflammation in Knee Osteoarthritis." Cartilage: 1947603520958149.

Varma, V. R., S. Varma, Y. An, T. J. Hohman, S. Seddighi, R. Casanova, A. Beri, E. B. Dammer, N. T. Seyfried, O. Pletnikova, A. Moghekar, M. R. Wilson, J. J. Lah, R. J. O'Brien, A. I. Levey, J. C. Troncoso, M. S. Albert and M. Thambisetty (2017). "Alpha-2 macroglobulin in Alzheimer's disease: a marker of neuronal injury through the RCAN1 pathway." Mol Psychiatry **22**(1): 13-23.

Wang, Q., C. Yu, S. Shi, X. Su, J. Zhang, Y. Ding, Y. Sun, M. Liu, C. Li, X. Zhao, W. Jiang and T. Wei (2019). "An analysis of plasma reveals proteins in the acute phase response pathway to be candidate diagnostic biomarkers for depression." Psychiatry Res **272**: 404-410.

Wang, X., K. Wong, W. Ouyang and S. Rutz (2019). "Targeting IL-10 Family Cytokines for the Treatment of Human Diseases." Cold Spring Harb Perspect Biol **11**(2).

Weber, K. T., S. Satoh, D. O. Alipui, J. Virojanapa, M. Levine, C. Sison, S. Quraishi, O. Bloom and N. O. Chahine (2015). "Exploratory study for identifying systemic biomarkers that correlate with pain response in patients with intervertebral disc disorders." Immunol Res **63**(1-3): 170-180.

Supplementary text #2

Willis, C. R., A. Seamons, J. Maxwell, P. M. Treuting, L. Nelson, G. Chen, S. Phelps, C. L. Smith, T. Brabb, B. M. Iritani and L. Maggio-Price (2012). "Interleukin-7 receptor blockade suppresses adaptive and innate inflammatory responses in experimental colitis." J Inflamm (Lond) **9**(1): 39.

Won, J. C., C. Y. Park, S. W. Oh, E. S. Lee, B. S. Youn and M. S. Kim (2014). "Plasma clusterin (ApoJ) levels are associated with adiposity and systemic inflammation." PLoS One **9**(7): e103351.

Wåhlén, K., M. Ernberg, E. Kosek, K. Mannerkorpi, B. Gerdle and B. Ghafouri (2020). "Significant correlation between plasma proteome profile and pain intensity, sensitivity, and psychological distress in women with fibromyalgia." Sci Rep **10**(1): 12508.

Wåhlén, K., B. Ghafouri, N. Ghafouri and B. Gerdle (2018). "Plasma Protein Pattern Correlates With Pain Intensity and Psychological Distress in Women With Chronic Widespread Pain." Front Psychol **9**: 2400.

Wåhlén, K., P. Olausson, A. Carlsson, N. Ghafouri, B. Gerdle and B. Ghafouri (2017). "Systemic alterations in plasma proteins from women with chronic widespread pain compared to healthy controls: a proteomic study." J Pain Res **10**: 797-809.

Yingchun, H., M. Yahong, W. Jiangping, H. Xiaokui and Z. Xiaohong (2018). "Increased inflammation, endoplasmic reticulum stress and oxidative stress in endothelial and macrophage cells exacerbate atherosclerosis in ApoCIII transgenic mice." Lipids Health Dis **17**(1): 220.

Zamanian Azodi, M., M. Rezaei Tavirani, A. Arefi Oskouie, M. Hamdieh, M. K. Derakhshan, A. Ahmadzadeh, F. Zayeri, N. Nejadi, M. Rezaei Tavirani, V. Mansouri, M. Rostami-Nejad and R. Vafaee (2017). "Fluoxetine Regulates Ig Kappa Chain C Region Expression Levels in the Serum of Obsessive-Compulsive Disorder Patients: A proteomic Approach." Iran J Pharm Res **16**(3): 1264-1271.

Zewinger, S., J. Reiser, V. Jankowski, D. Alansary, E. Hahm, S. Triem, M. Klug, S. J. Schunk, D. Schmit, R. Kramann, C. Korbel, E. Ampofo, M. W. Laschke, S. R. Selejan, A. Paschen, T. Herter, S. Schuster, G. Silbernagel, M. Sester, U. Sester, G. Assmann, R. Bals, G. Kostner, W. Jahnen-Dechent, M. D. Menger, L. Rohrer, W. Marz, M. Bohm, J. Jankowski, M. Kopf, E. Latz, B. A. Niemeyer, D. Fliser, U. Laufs and T. Speer (2020). "Apolipoprotein C3 induces inflammation and organ damage by alternative inflammasome activation." Nat Immunol **21**(1): 30-41.

Zhao, J., Y. X. Fan, Y. Yang, D. L. Liu, K. Wu, F. B. Wen, C. Y. Zhang, D. Y. Zhu and S. Zhao (2015). "Identification of potential plasma biomarkers for esophageal squamous cell carcinoma by a proteomic method." Int J Clin Exp Pathol **8**(2): 1535-1544.

Zhu, Y., S. Han, H. Zhao, J. Liang, J. Zhai, Z. Wu and G. Qiu (2011). "Comparative analysis of serum proteomes of degenerative scoliosis." J Orthop Res **29**(12): 1896-1903.

Zou, Y., Y. Wang, S. B. Wang, Y. G. Kong, Y. U. Xu, Z. Z. Tao and S. M. Chen (2016). "Characteristic expression and significance of CCL19 in different tissue types in chronic rhinosinusitis." Exp Ther Med **11**(1): 140-146.
