# Supplementary material for: Thermal Pain Thresholds Are Significantly Associated with Plasma Proteins of the Immune System in Chronic Widespread Pain—An Exploratory Pilot Study Using Multivariate and Network Analyses
Source: J Clin Med. 2021 Aug 18;10(16):3652. doi: 10.3390/jcm10163652 (PMC8396857; doi:10.3390/jcm10163652)
Supplement: Supplementary file 1 [file jcm-10-03652-s001.zip › supplementary material _ two files/Supplementary text#1 final.pdf]

Supplementary text #1

Regression of CPT an HPT using clinical variables as regressors

CON

The obtained regressions were not significant according to CV-ANOVA.

In detail, for the non-significant CPT regression (one predictive component; R^2=0.25, Q^2=-0.07, CV-ANOVA p-value: ns) the most important variables (VIP>1.0) were HADS (VIPpred=1.39; p(corr)=0.79) and age (VIPpred=1.06; p(corr)=-0.60) followed by PCS (VIPpred=0.88; p(corr)=0.50), QOLS (VIPpred=0.79; p(corr)=-0.45), and BMI (VIPpred=0.74; p(corr)=0.42).

For the non-significant regression of HPT (one predictive component; R^2=0.11, Q^2=-0.13, CV-ANOVA p-value: ns) were found that age (VIPpred=1.62; p(corr)=0.89) and PCS (VIPpred=1.20; p(corr)=-0.66), were the most important (VIP>1.0) followed by QOLS (VIPpred=0.77; p(corr)=0.42), HADS (VIPpred=0.58; p(corr)=-0.32) and BMI (VIPpred=0.05; p(corr)=-0.02).

CWP

The regression of CPT was not significant. In detail, for the non-significant CPT regression (R^2=0.38, Q^2= 0.06, CV-ANOVA p-value: ns; one predictive component) HADS (VIPpred=1.47; p(corr)=0.78) and QOLS (VIPpred=1.25; p(corr)=-0.67), NRS-neck (VIPpred=1.04; p(corr)=0.56) were the most important (VIP>1.0) followed by age (VIPpred=1.00; p(corr)= -0.53), NRS-shoulders (VIPpred=0.88; p(corr)= 0.47), PCS (VIPpred=0.60; p(corr)=0.32) and BMI (VIPpred=0.21; p(corr)=0.11).

For the significant regression of HPT (R^2=0.67, Q^2= 0.41, CV-ANOVA p-value: 0.043; one predictive component) were found that HADS (VIPpred=1.78; p(corr)=-0.85), QOLS (VIPpred=1.60; p(corr)=0.77), were the most important and significant variables (VIP>1.0) followed by BMI (VIPpred=0.81; p(corr)=-0.39), NRS-shoulders (VIPpred=0.48; p(corr)=-0.23), NRS-neck (VIPpred=0.43; p(corr)=-0.21), PCS (VIPpred=0.34; p(corr)=0.16) and age (VIPpred=0.27; p(corr)=0.13).
